# Photoactive Cements: A Review

**DOI:** 10.3390/ma15155407

**Published:** 2022-08-05

**Authors:** Dominika Dudek, Magdalena Janus

**Affiliations:** Department of Environmental Engineering, Faculty of Civil and Environmental Engineering, West Pomeranian University of Technology in Szczecin, al. Piastów 50, 70-311 Szczecin, Poland

**Keywords:** cement, photocatalytic activity, cementitious composites, mechanical properties

## Abstract

This article presents a short overview of modified cements with photocatalytic activity. First, the types and three main methods of obtaining photoactive cements are presented. The most frequently used modification method is the incorporation of a photocatalyst into the total mass of the cement. The second group analyzed is cements obtained by applying a thin layer of photoactive materials, e.g., paints, enamels, or TiO_2_ suspensions, using various techniques. The third group is cement mortars with a thick layer of photoactive concrete on the top. In addition, methods for determining the photoactivity of cement composites, mechanical properties, and physicochemical parameters of such materials are briefly presented. Finally, examples of investments with the use of photoactive cements and development prospects are shown.

## 1. Introduction

Today, it would be difficult to imagine the image of modern buildings and architecture without concrete, which is a widely used construction material. Nevertheless, it is not only the functionality, aesthetics, and durability of buildings that justify the use of this composite material. Currently, an important argument in favor of its use is its contribution to the protection of the natural environment [1,2]. Recently, self-cleaning special cements have become more and more popular, which are the basic component of ecological concrete products, architectural and facade concrete, and precast concrete products, as well as construction chemicals and cement mortars. The biggest problem regarding air quality around large urban areas is that the air exceeds the permissible standards for the content of NOx, SO_2_, and other hazardous substances in the air, mainly caused by the rapid development of the automotive industry. Photoactive cements that break down gaseous pollutants in an environmentally friendly manner come to the rescue. The photocatalytic material used for its modification, most often titanium dioxide TiO_2_, is responsible for the photoactivity of such cements [1,2,3,4]. The addition of nanometric TiO_2_ to cement was first registered in Japan in the early 1990s [5]. Currently, photocatalytic cements are used on a large scale in the production of building materials, concrete, and paving stones covering sidewalks and streets where we walk.

## 2. Mechanism of Photocatalysis, Types of Photoactive Cements and Their Preparation

(a) Photocatalytic mechanisms of TiO_2_
  Suitable semiconductors are photocatalysts capable of carrying out the photocatalysis process. The most famous semiconductor in the world is titanium dioxide. The supply of energy in the form of light to the semiconductor causes the electrons from the base band to jump to the conduction band, and oxidation and reduction reactions take place in both bands [1]. The mechanism of photocatalytic oxidation is well understood and generally comprises four major steps [6,7,8,9]:
  (1) Adsorption of reagents on the surface of the photocatalyst,
  (2) Excitation of the photocatalyst with UV radiation and photo-generation of e--h+ pairs (photo-inducted electrons and holes),
  (3) Generation of hydroxyl radicals from water molecules adsorbed on the semiconductor surface,
  (4) Generated hydroxyl radicals and other structures take part in decomposition of such compounds as organic compounds, nitrogen oxides and other compounds,

(b) Types of photoactive cements.

In the literature, the most frequently modified types of cement are ordinary Portland cement (OPC), white cement (WC), and aluminum-calcium cement (CAC); slag and fly ash are less frequently used binders, because according to Jimenez-Relinque [6], the addition of ash and slag reduces the efficiency photocatalytic effect of photoactive cement. According to Jin [7] et al., CAC increases the NOx absorption capacity and shows more permanent binding of these gases in aluminum-containing phases. Moreover, a relatively new interest of scientists is polymer-cement composites, which are obtained by mixing various types of cements with polymers [8,9]. For example, Baltes et al. [8] mixed CAC with polyvinyl alcohol (previously mixed with glycerol) to obtain a mortar. However, it was found that the solution does not guarantee resistance to the aggressive environmental conditions affecting the material; therefore, they additionally created a coating improving the water resistance. They obtained it by mixing a commercial silica-based enamel with TiO_2_ nanopowder [8]. On the other hand, Zhao et al., for the modification of the polymer-cement coating, used sulfaluminum cement (SAC), which, compared to Portland cement, is characterized by lower alkalinity and permeability, and thus better durability [9]. Guo and other researchers [10] used TiO_2_ nanoparticles mixed with epoxy resin polymers to modify the Portland cement mortar, which improved the mechanical properties and flowability of the composite.

**Figure 1:**
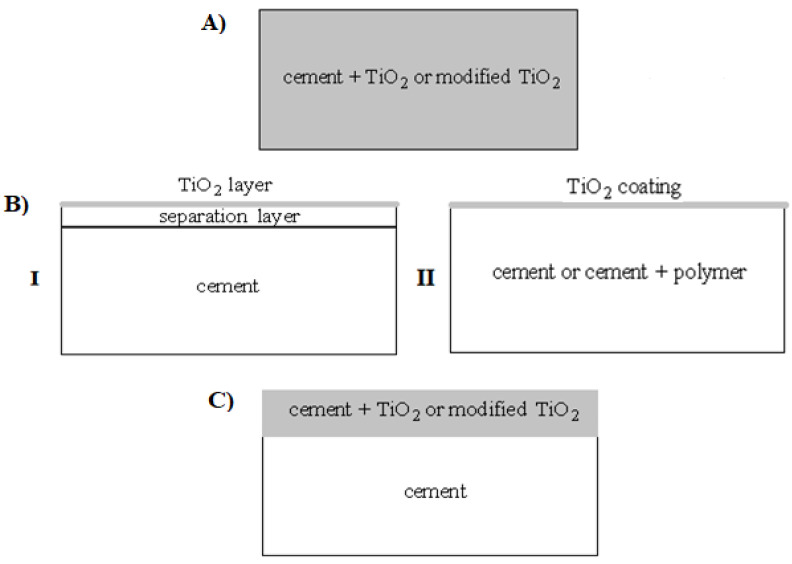
Scheme of possible methods of cement modification by photocatalysis: (**A**) incorporation method, (**B**) surface treatment method: with (I) and without (II) a separation layer, (**C**) surface coating with a composite cement surface layer containing the photocatalyst.

Generally, the literature provides three technologies for obtaining photoactive cements, which are presented in the example of the most commonly used metal oxide for modification—titanium dioxide, TiO_2_ (Table 1, Figure 1). The first and most frequently used method of modification of cements is the incorporation method, which consists of making a cement material containing the photocatalyst in its entire mass (Figure 1A). The second group is cements obtained by the surface treatment method, with a thin layer of photocatalyst on the outside: we distinguish two variants of this modification, including: (I) coating the separation layer; or (II) direct coating of the cement with a thin layer of photocatalyst (Figure 1B). The third group of photoactive cements is materials obtained by incorporating the photocatalyst only into the top layer of the composite (surface coatings with a composite cement surface layer containing the photocatalyst) (Figure 1C).

### 2.1. Obtaining Photoactive Cements by Incorporation Method

(a) Examples of preparation of the cements by intercalation method using TiO_2_

The first method of obtaining cement composites consists of adding various amounts of TiO_2_ or replacing a part of the cement in the mixture (cements modified in mass with TiO_2_) in the form of a photocatalyst suspension or dry powder and requires only simple mixing of both materials [11,12]. It is known that heterogeneous photocatalysis is a process that takes place only on the surface of the material. Therefore, the use of the method of incorporating the photocatalyst in the entire mass limits the photocatalytic efficiency of such material because the sun’s rays do not reach the deeper layers of cement [13,14]. On the other hand, there were reports in the literature about the improvement of the mechanical properties of cements by additional filling of the pores and the interaction of TiO_2_-cement components. Therefore, from the point of view of the durability of the material in a long period of use, the admixture of a photocatalyst seems to be a better solution [3,8,11]. Moreover, most of the research work focuses on commercial TiO_2_ as an additive to cement materials. It is known that both the size of the photocatalyst particles and the type of binder used for the production of the photoactive cement are responsible for the photocatalytic properties. Jimenez-Relinque et al. [6] designed photocatalytic cement mortars containing 2% TiO_2_ P25 as an additive per cement mass. The samples were made with the use of various types of binders: Portland cement, aluminum-calcium cement, blast furnace slag, and fly ash. They observed that cement mortars had photocatalytic activity, but cement matrices containing commercial Portland cement and aluminum-lime cement were more active, while ash and slag mortars turned out to be the least active. On the basis of their research, they found that the composition of the cement matrix affects the value of the redox potential of the water phase in the pores and the photoabsorption energy [6]. In turn, Folli et al. [12] investigated the effect of TiO_2_ photocatalyst particle size on the photocatalytic activity of cement slurries. They obtained cement samples by dry-mixing fresh Portland white cement powder with 3.5% commercial microsized m-TiO_2_ and nanosized n-TiO_2_, both containing 100% anatase. To compare the photocatalytic activity of the obtained cements, they used the organic dye degradation test, rhodamine B (RhB), under the influence of ultraviolet radiation. They reported that m-TiO_2_ shows greater efficiency of dye decomposition in the cement environment, which is probably associated with a greater degree of dispersion and a larger area for dye adsorption. In addition, another study was carried out at the University of Milan in Italy, using m-TiO_2_ and n-TiO_2_ powders [15]. The results of these studies showed that dispersion and distribution are more convenient when using m-TiO_2_, as the latter shows a significant tendency to agglomerate and thus works towards reducing the total reactive surface available for initiating photocatalytic reactions. It was also found that the use of n-TiO_2_ was more advantageous since the composite had a better ability to adsorb contaminants that could readily permeate the agglomerates formed. The pros and cons of such a solution are also presented in Section 2.1. (b). Returning to the binder effect, Chen and Poon [16] compared the photocatalytic activity of Portland (OPC) and white (WC) cement, where the amount of additional admixture of photocatalysts (P25 or anatase) was 5% and 10% of the total cement mass. They noticed that the samples prepared with WC showed better photocatalytic activity than those prepared with OPC, due to its gray color caused by metallic additives. This was confirmed by studies conducted by Guo et al. [17], as they found that the poorer performance of OPC mixtures with nano-TiO_2_ (2% and 5% by weight of cement) in photocatalytic NOx removal is due to the combination of stronger light absorption and lower charge separation caused by OPC. Cassar et al. [18] mixed TiO_2_ with white cement (5% by weight of cement) and compared the photocatalytic activity of such a cement mortar without the addition of TiO_2_. They found that WC without the addition of TiO_2_ also exhibits photocatalytic activity due to the presence of photocatalytic oxides in its matrix. In turn, Lee et al. [19] obtained photoactive cement samples via 5% and 10% TiO_2_ replacement by mass of cement. In order to incorporate the photocatalyst nanoparticles into the cement matrix, they used a preprepared aqueous suspension of commercial TiO_2_ P25 containing 80% anatase and 20% rutile as an additive to commercial Portland cement (OPC). They investigated the photocatalytic activity of the obtained cements containing TiO_2_ nanoparticles in terms of the degradation of NO and NO_2_ gases. Their main observation was the fact that the photocatalytic activity of the obtained cement pastes in removing NOx under the influence of UV light was very similar, while in the absence of UV light, they observed a greater ability to bind NO_2_ than NO by the cement matrix, which proves that OPC is a binder designed to bind NOx, especially NO_2_.

(b) Examples of preparation of the cements with modified TiO_2_

The use of modified titanium dioxide is a procedure aimed at improving photocatalytic efficiency, reducing production costs, and enhancing the photocatalytic activity of cement materials obtained by intercalation. J. K. Ratan et al. [13] believe that the use of m-TiO_2_ reduces the photocatalytic activity of the self-cleaning cement compared to the more expensive n-TiO_2_. However, the use of a more active photocatalytic n-TiO_2_ in a photoactive cement formulation has many advantages, including a larger surface area, a lower band gap, and a reduced recombination rate, as well as commercial disadvantages. n-TiO_2_ can cause serious health problems in humans as a result of inhalation of nanoparticles during the production and use of self-cleaning cement [20]. Moreover, the previously mentioned obstacle in commercialization of the manufactured product is also its high cost. The third disadvantage leading to the deterioration of the performance of the self-cleaning cement is the fact that the n-TiO_2_ shows a greater degree of agglomeration in the cement matrix. Therefore, in order to overcome the above-mentioned drawbacks of using n-TiO_2_, scientists are making considerable efforts to improve the photocatalytic performance of m-TiO_2_-based self-cleaning cements, which solves the problems. Summing up, the research carried out so far on the influence of particle size distribution on the performance of the cement matrix has not revealed a decisive direction that should be followed. From the point of view of the distribution of contaminating particles, it would be more reasonable to use n-TiO_2_, because it exhibits a more effective decomposition of nitrogen oxides as well as superhydrophilicity, reducing the contact angle of water and creating a homogeneous hydrophilic film of the modified substrates [21]. It is also worth emphasizing that scientists often look for more economical solutions. For example, J. K. Ratan et al. [13], in order to increase the activity, proposed introducing a co-adsorbent in the form of calcined dolomite into the cement matrix, together with m-TiO_2_. A cement sample prepared by adding 10% calcined dolomite (sedimentary rock associated with limestone) to a mixture of 87% clinker and 3% micronized TiO_2_ showed a significant improvement in photocatalytic activity resulting from increased light energy absorption and methylene blue adsorption [13]. As mentioned earlier, modified titanium dioxide is increasingly used to improve the properties of the cement matrix. Similar to the previously described solution, Saini et al. [4] proposed to obtain self-cleaning cement by uniformly mixing 87% clinker and 10% calcined dolomite, with the difference that a 3% addition of surface-fluorinated m-TiO_2_ was used for modification. The self-cleaning cement modified in such a ready state showed amazing self-cleaning properties and ensured a remarkable degradation of NO_X_ from the air [4]. Amor et al. applied a 0–20 wt.% of double lamellar hydroxides based on ZnO, Al_2_O_3_, and TiO_2_ for the clinker paste, which also ensured better self-cleaning properties [22]. On the other hand, Tyukavkina et al. [3] mixed powders of titanium dioxide obtained from the waste sorbent generated during the treatment of heavy non-ferrous metal discharges with Portland cement. They showed that the modified TiO_2_ acts as a modifier, accelerating the cement hydration and hardening processes and acquiring the ability to self-clean thanks to the photocatalytic activity of the added additive [3]. In the literature, there is also the use of nitrogen-modified TiO_2_ (TiO_2_/N) as an additive in the amount of 1, 3, and 5 wt.% to Portland cement [23], modified with carbon and nitrogen (TiO_2_-N,C) in the amount of 10 wt.% and 20 wt.% [24] in order to provide photocatalytic properties.

(c) Examples of the preparation of cements with the addition of another type of photocatalyst

In general, titanium dioxide is most often used to impart photocatalytic activity to cements, which means that its solar-induced photocatalytic efficiency is probably sufficient. Moreover, the number of literature reports on the use of a different type of photocatalyst is relatively small and it constitutes a small percentage of research. An interesting solution proposed by Moreira et al. [25] is the use of red mud (RM) and Nb_2_O_5_ as an additive in the amount of 2 wt.%, each in relation to Portland cement. The proposed solution has the potential to be used in self-cleaning surfaces. Both of the mortars showed the ability to photodegrade methylene blue. It is also worth emphasizing that red mud is an alkaline waste material in the process of bauxite enrichment in order to produce alumina [26]. Therefore, its addition to cement is an environmentally friendly method of waste disposal, consistent with the principles of sustainable development [27]. In studies by Geng et al. [28], two different BiOBr precursor solutions were used to modify white cement pastes, obtained by dissolving bismuth(III) nitrate(V) · 5H_2_O in the amount of 1.5% by weight, based on the weight of the cement in ethylene glycol or nitric acid. The solutions were then added dropwise to an appropriate amount of water and NaBr. It was found that the microstructure of the obtained cement slurries was dominant in increasing the photocatalytic activity [28]. A new direction of research has become non-metallic photocatalysts that react to visible light, which can be used in areas not exposed to sunlight for air purification, such as underground parking lots and tunnels. In the latest research by Yang et al. [29] for the modification of Portland cement, the researchers used a suspension of polymeric carbon nitride (PCN) powder, which was mixed with cement in the weight ratio of PCN to cement, respectively, 0%, 0.1%, 0.5%, 1%, and 2%. The results of the research on the phase composition and microstructure of the cement matrix showed that PCN can play the role of a micro-filler, improving the microstructure, reducing porosity, and hindering the diffusion of ions and water in cement systems [29].

### 2.2. Preparation of Photoactive Cements by Surface Modification

(a) Formation of thin TiO_2_ films with/without a separation layer

The second type of modification is the coating of cement mortars with a thin layer of photocatalyst. According to Wang et al. [30], the use of surface treatment technology reduces costs due to the reduced need for a photocatalyst. Additionally, compared to the incorporation method, the application of surface treatment technology has a wider scope because it is possible to impart photocatalytic properties to already existing buildings.

However, the solution of enriching the surface of the cement mortar is limited by the poor adhesion of the photocatalytic material to the substrate, which causes the catalyst to fall off during use, thus reducing the photocatalytic efficiency [28,31]. A valuable chemical substance used to prevent the release of TiO_2_ particles from the cement surface into the environment is SiO_2_, which exhibits high pozzolanic activity with cement [32,33]. Wang et al. [31] coated the surface of white Portland cement with the prepared SiO_2_/TiO_2_ composite by depositing TiO_2_ on SiO_2_ (I) spheres. They found that SiO_2_ improves the photocatalytic activity to some extent, due to the larger surface for adsorption of pollutants and the adhesion between the coating and the cement. However, Mendoza et al. [34] covered the cement surface by a spraying method with four different commercial TiO_2_ suspensions from Cristal Global and a self-made stable titanium suspension obtained by hydrolysis in the acid medium of tetraethyl orthosilicate (TEA). In their work, they proposed an intermediate layer (II) between the photocatalytic coating and the substrate. They covered the cement first with a TEA suspension, and then they covered the dried cement samples with the above-mentioned photocatalytic suspensions of TiO_2_. Their research showed that the separation layer between the substrate and the coating did not effectively stabilize TiO_2_, but good adhesion was observed when jointly applied to TEA, probably because of the interactions encountered between SiO_2_ and TiO_2_ sols [34].

On the other hand, Guo et al. [35] focused on comparing the effectiveness of the intercalation method and the method of creating thin films. Concrete samples were obtained by mixing Portland cement, fly ash, water, and recycled glass in appropriate proportions. In the case of the nano-TiO_2_ (P25, Degussa) mixing method, 5% by weight of the cement materials was added to the cement mortar mix. In turn, in the case of the surface treatment method, they sprayed the n-TiO_2_ suspension in ethanol directly on the concrete surface and found that the sample obtained with this method was more effective in removing NOx and was characterized by significant abrasion resistance [35].

(b) Forming thin films with use of modified TiO_2_

The use of surface treatment technology can be a special approach, increasing the resistance and durability of cement-based materials in relation to the external environment affecting them. The solution proposed by Vulic et al. [36], which improves the photochemically induced self-cleaning properties: dye discoloration and photo-inducted hydrophilicity, and lowers mortar systems surface roughness, is the coating of cement with a TiO_2_/ZnAl suspension based on layered double hydroxides associated with TiO_2_. Another approach proposed by Wang et al. [37] is to coat the cement paste with a hydrophobic TiO_2_ coating obtained in a solvothermal process to obtain a core-shell structure prepared using benzoic acid as a surfactant. Hydrophobic TiO_2_ showed about 3.6 times the efficiency in removing organic dye compared to the untreated cement paste. Benzoic acid has a surface bond formed with TiO_2_, which leads to the hydrophobicity of TiO_2_, thanks to which the surface prevents water penetration, supports its durability, and supports the self-cleaning effect [37].

A promising approach is also to combine the advantages of a superhydrophilic surface (lamellae formed instead of water droplets are able to easily remove decomposed compounds) with a hydrophobic impregnation, which, on the one hand, protects the surface from the harmful effects of oil contamination. On the other hand, it prevents water from penetrating into the porous structure of the substrate. Such a solution was proposed by Carrascosa et al. [38] by simply spraying a sol containing titanium nanoparticles, a silica oligomer ensuring good adhesion to the substrate, and a hydrophobic agent—polydimethylsiloxane. Due to the effective penetration of the product into the porous structure of concrete and the formation of a homogeneous and dense coating on the surface, the prepared silica matrix showed long-term resistance to external conditions. Moreover, after exposure to sunlight, an underwater superoleophobic effect was noted, which can be used to remove oil pollution without the need for detergents [38]. Another interesting example of the use of modified TiO_2_ is the studies in which Feng and Li [39] covered samples of Portland cement with a suspension of TiO_2_/g-C_3_N_4_ powder in polyethylene glycol (0.01%, 0.1%, and 1% in mass) with a spraying method for different times (1–3 times). It was found that the cement slurry with 1% content of TiO_2_/g-C_3_N_4_ in suspension and 3-times spraying had the best photocatalytic efficiency. Moreover, the thus-obtained cement paste may be very promising in the large-scale water treatment process, as it showed excellent degradation efficiency of methylene blue (~98%), even after five cycles [39].

(c) The formation of coatings using a different type of photocatalyst

It is known from the literature that TiO_2_ is the most frequently used semiconductor particle to produce cements with photocatalytic activity [1]. The advantages of its use are low cost, chemically stable nature, and no toxicity [40]. However, scientists also started looking for other photocatalysts that would be active in visible light, as TiO_2_ with a wide bandgap (3.2 eV) cannot use visible light, as it only absorbs UV light (λ ≤ 387 nm). For example, Zhong et al. [41] covered the surface of white cement with a suspension of carbon nitride graphite composite, previously mixed with an appropriate amount of nanosilica (gC_3_N_4_-SiO_2_), and found an improvement in the activity of the material in visible light.

### 2.3. Installation of the Photocatalyst Only in the Top Layer of Photoactive Cement

(a) Examples using TiO_2_ and modified TiO_2_

The third method of obtaining photoactive cements is the addition of a photocatalyst only to the outer layer of the composite. The method is successful because in most cases it is not necessary to enrich the entire mass of the cement element with the photocatalyst. It has been proven that the coating layer made of a composite material with photocatalytic properties is sufficient to increase the durability of the element and obtain the photocatalytic effect [33,42,43].

For example, Hernández-Rodríguez et al. [42] imparted photoactivity by incorporating Aeroxide TiO_2_ P25 catalyst (Evonik Industries, Essen, Germany) into the one-centimeter surface layer of cement samples (5 and 10 wt.%). On the basis of the research, they found that the addition of the photocatalyst does not significantly affect the mechanical properties of the cement mortar, but it accelerates the setting time. Lee et al. [33] used a photocatalyst obtained by coating TiO_2_ with SiO_2_, using the sol-gel method as an additive in the amount of 4 wt.% for the production of an eight-millimeter top layer of concrete blocks [33]. In turn, Binas et al. obtained photoactive cement coatings from commercially available powders by adding 5% *w/w* and 10% *w/w* of TiO_2_ photocatalyst with Mn admixture and found that the greater the addition of the photocatalyst to the coating (higher concentration), the better the activity [11].

(b) Example using a different type of photocatalyst

A relatively new and worth considering trend is the use of a different type of photocatalyst to modify the top layer of cement composites. For example, Huang et al. [44] prepared a cement mortar with a thin layer of mortar containing the addition of g-C_3_N_4_/CoAl-LDH nanocomposite obtained by co-precipitation. Firstly, they prepared the cement mortar sample with water/cement/standard sand at the mass ratio of 0.5:1:3. Then, they obtained a fresh cement paste mixed with 0.25 wt.% g-C3N4/CoAl-LDH (w/c = 0.5), which was spread on the cut face of prepared mortar with a thickness of around 5 mm. The obtained cement nanocomposite showed better photocatalytic parameters than pure photocatalyst.

## 3. Test Methods Used for the Evaluation of the Photocatalytic Activity of Cements

**Figure 2:**
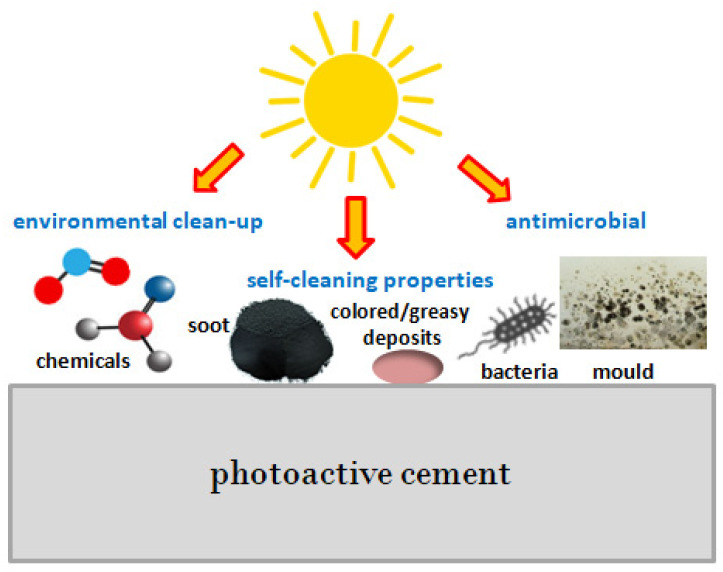
Schematic diagram of multi-functional photoactive cements (based on [49]).

Despite the enormous interest in photocatalytic cements and the materials obtained from them, a need arose to determine the photocatalytic activity in order to control the quality and compliance with specifications [45,46]. Currently, there is no standard procedure for testing and determining the photocatalytic activity of such materials [1]. The freedom of choice of methods limits and presents a challenge in comparing the photocatalytic activity and efficiency of cementitious materials from different sources [47]. Moreover, the existing standards do not take into account factors influencing the results, such as the surface diversity of photoactive cements, e.g., their roughness, pore structure, and sample size, as well as differences in experimental conditions, e.g., radiation source and intensity, gas flow rate, temperature, and relative humidity; this makes it difficult to compare the results [48,49]. Photoactive cements are most often tested for their suitability for specific purposes, including tests involving the purification of the surrounding environment—removal of toxic gases (e.g., NOx, SOx, VOCs) from the air, tests determining the self-cleaning ability (without the use of manual work and detergents), and antimicrobial activity (Figure 2) [50].

(a) Photoactive tests used for determining the photocatalytic efficiency by air purification

Test methods for determining the photocatalytic efficiency by removing environmental pollutants are carried out by reducing the amount of gases, which can be divided according to the type of pollutant: NOx, SOx, CO, and VOCs (volatile organic compounds), e.g., BTEX tests (in ppb levels). According to the researchers, the nature of the cement matrix is particularly suitable for the degradation of gaseous pollutants [4,51].

In the case of determining the photocatalytic performance of photoactive cements, the most frequently used method is the NOx degradation test under the influence of ultraviolet radiation. The main reason for the use of the NOx removal test among studies on the commercialization of photocatalytic cement products is that these gases are produced in large amounts as byproducts of incomplete combustion of gasoline and fossil fuels, reducing air quality around highly congested places [1,4,34,46]. Air purification tests are most often carried out in accordance with ISO 22197-1 [34,52]. According to this method, a sample with dimensions of 5 cm × 10 cm and a thickness of usually 5 mm is placed in an inert photoreactor, where it is activated with UV-A radiation of the appropriate intensity. Moreover, it is exposed to humid (RH = 50% at 25 °C) air and dry NO at a certain time. Finally, the exhaust gas stream is directed to the NOx chemiluminescent analyzer [45,53,54]. Test parameters in accordance with the ISO 22197-1 standard are given in Table 2 [55].

Commonly used test methods are dynamic and static NOx reduction tests [1]. In a dynamic test, which is a better reflection of real conditions, NO or both NO and NO_2_ gases are mixed with air and are then introduced into the sample chamber at a constant flow rate, in contact with its surface. The resulting gas concentration is measured continuously in real time in order to compare the input and output gas concentrations [56]. In turn, in the static method, a certain amount of NO gas is introduced into the photoreactor and recirculated in a closed circuit, which means that no air exchange takes place during the test; therefore, it is believed that the dynamic method better represents the real conditions [1]. It is also worth noting that the photocatalytic activity of the samples is influenced by the relative humidity. The increase in humidity causes the NO removal rate to decrease [57,58]; so, to prevent interfering with the measurement results, the samples are dried before the test in an oven, for example, at 45 °C for 48 h [56].

It is also worth mentioning here the studies by Jimenez-Relinque and Castellote [45], who proposed a new method for assessing the photocatalytic efficiency of cement materials based on tetrazolium nitro blueite (NBT). They reported that the NBT indicator is a promising alternative to the above-described conventional NOx removal method due to its versatility, low cost, much shorter duration of the in situ test (only 10 min), easily noticeable color change (from yellow to blue), and applicability test on porous, rough, and colored surfaces [1,45]. In addition to NOx, the pollutants to be removed with photocatalytic cement mortars are volatile organic compounds (VOCs). To study the efficiency of their removal, tests were started to quantify the photodegradation of a mixture of VOCs representative of atmospheric pollution, abbreviated as BTEX (benzene, toluene, ethylbenzene, o, m, p-xylenes) [1,46]. BTEX tests for the photodegradation of organic compounds are carried out in accordance with ISO 22197-3 [59] using a specially designed flow reactor with a stirrer ensuring the same gas concentration in the air. For example, Martinez et al. [60] investigated the photoactivity of photocatalytic coatings containing 10% (*w/w*) TiO_2_ in a polymer matrix applied to Portland cement mortars. They conducted experiments using the following analysis parameters: in various concentration ranges of BTEX at the inlet (260–2600 ppbV BTEX), flow rate (0.1–1.5 L/min), with UV-A illumination (6 W/m^2^), and relative humidity: 0–90% [60]. On the basis of the research, they found that the effectiveness of the photocatalytic removal of BTEX depends mainly on the humidity in the absorbent places, the adsorption capacity of pollutants in these places, and the concentration of pollutants.

(b) Photoactive tests used for determining the self-cleaning properties

Self-cleaning properties obtained by introducing photocatalytic properties into cements prevent the accumulation of pollutants in the form of visible stains on the external parts of building structures [4]. This is achieved by exposing the cement surface to sunlight, which makes the surface hydrophilic due to photons. Self-cleaning cement, generating reactive oxygen species in the presence of photons, oxidizes available pollutants to water, carbon dioxide, and less unpleasant byproducts. The literature [61] shows that TiO_2_ nanoparticles themselves lose their hydrophilicity as soon as the UV radiation exposure ceases; however, in combination with SiO_2_ in the cement mortar, the UV activation effect is extended in the conditions of complete absence of UV radiation (in the dark) [62]. In addition, the porous structure of the hardened cement is directly designed for the effective contact of pollutants with the photocatalyst, facilitating photo-oxidation of the pollutants.

In order to test the self-cleaning properties, tests are conducted for the decomposition of organic dyes, such as rhodamine B [2,38,63,64,65,66,67,68,69,70,71,72,73], methylene blue [22,39,74,75,76,77,78,79], and methyl orange [74,80,81,82]. Malachite green [83] and Congo red [84] are used much less frequently for tests.

Colorimetric tests are most often carried out in accordance with the Italian standard UNI 11259, according to which a material is considered photocatalytic if the change in color intensity decreases by more than 20% after 4 h and 50% after 24 h of exposure to UV-A light. Rhodamine B is used as the reference substance, and its degradation is taken as an indicator of the self-cleaning capacity [54]. The method does not require complicated research equipment; however, the correct assessment of the self-cleaning efficiency is limited by the rough and uneven surface of the cements, which prevents the even distribution of the dye [45]. The rhodamine technique is also not suitable for the photocatalytic evaluation of cements dyed in a similar color to the dye itself, because it is difficult to notice any changes; moreover, it cannot be the main method of photocatalytic evaluation of cements due to possible interaction with organic admixtures in the products [46]. In connection with the previously mentioned problems, Jimenez-Relinque and Castellote [49] proposed the use of a fluorescent terephthalic acid probe, which provides direct monitoring of photocatalytic oxidation regardless of the specific pollutant, and also enables the examination of colored, porous, and rough samples. In addition, the test is accurate and fast as it takes approximately 20 to 40 min to complete. Recently, semi-quantitative methods that use reducing pigments such as resazurin (Rz) have also been proposed [85,86,87]. The main advantages of these methods are low cost, simplicity, speed (the test takes about 20–30 min), and the possibility of application on colored materials due to the visible color reaction [45]. Another standardized method used to evaluate the photocatalytic efficiency of decomposition of organic compounds by photoactive cements is the methylene blue (MB) decomposition method (ISO 10678). According to some authors, this test is more appropriate for the assessment of water purification efficiency than for self-purification [45,88,89]. However, in the case of photoactive cements, this method serves both. On the one hand, in order to determine the water purification capacity, the test consists of immersing cured cement samples of appropriate dimensions in a certain amount and concentration of a methylene blue solution (pH about 6.5). First, the system is left in the solution for 24 h in the dark, then the sample is irradiated with UV-A radiation for 3 h, and the absorbance is monitored [13,25,65]. On the other hand, it is used to determine the self-cleaning ability of cement boards on the basis of the disappearance of the MB dye under the influence of light [4]. Then the cement slab is evenly covered with a specific amount of MB solution with a specific concentration. Then the coated plate is exposed to UV-A radiation, and the percentage of dye disappearance is monitored and quantified at regular intervals by measuring the absorbance of light [4].

In addition, in order to protect construction materials against the multiplication of microorganisms, photoactive cements have begun to be tested for antimicrobial properties that may increase their life, strength, and aesthetics [62]. The disinfecting properties of the self-cleaning cement prevent the growth of pathogenic microorganisms on its surface, thus increasing its life and aesthetic durability. Moreover, by inhibiting the multiplication of microorganisms, they protect people against harmful effects on their health [4,90,91]. Exposing the self-cleaning cement to sunlight generates electron-hole pairs, which in turn generate reactive oxygen species (ROS), causing photo-oxidation of cell membrane phospholipids and other cell components in order to inhibit the growth of microorganisms [92,93,94,95,96]. So far, studies on the assessment of the antimicrobial activity of cementitious materials have not been standardized [88]. Admittedly, there is an ISO 27447 standard [97], but it does not take into account the porous structure of photoactive cement materials [88]. This may be the main reason for a much smaller number of literature reports than in the case of the previously described methods of assessing photocatalytic activity. Nevertheless, according to the current state of knowledge, the antimicrobial activity of photoactive cements is tested against bacteria [4] and fungi (molds) [62]. For example, Saini et al. investigated the antimicrobial activity of clinker cement panels with a 10% addition of calcined dolomite and a 3% addition of surface-fluorinated m-TiO_2_ against Gram-positive *Bacillus subtilis*. It has been shown that the addition of modified m-TiO_2_ to the cement inhibits the growth of the bacteria used for the test [4]. Janus et al. [96] showed that the type of photocatalyst introduced into concrete slabs is a key factor influencing the rate of bacterial removal. The antibacterial properties of n-TiO_2_ were tested by inactivating *Escherichia coli* K12. Addition of a photocatalyst in the amount of 10 wt.% led to complete inactivation of bacteria used for tests under the influence of irradiation with visible light. Commercial n-TiO_2_ P25 and doped n-TiO_2_ (12 types) were used at different temperatures, i.e., 100, 300, and 600 °C. Moreover, the doped/modified n-TiO_2_ shows better antimicrobial properties compared to the commercially available n-TiO_2_. Hegyi et al. [62], in turn, investigated the ability to inhibit the growth of *Aspergillus niger* and *Penicillium notatum*, which are often found on building surfaces, both inside and outside buildings, and cause population diseases due to the release of aflatoxins harmful to health. They investigated the ability of mold inhibition by cement composites based on white Portland cement with the addition of nano-TiO_2_. They found that the concentration of nanoparticles in the amount of 5 wt.% TiO_2_ provides a better biocidal effect than cement with the addition of 6 wt.%, which was most likely due to the difficulty of uniformly dispersing a large amount of nanoparticles in the cement matrix. Moreover, it has been shown that the resistance to mold growth differs depending on its species, by comparing the behavior of composites with the same nano-TiO_2_ content when exposed in each of the two contaminating media.

It is also worth emphasizing here that soot is an important problem, as it settles on the surface of the building and reduces its aesthetic value. Therefore, scientists determine the efficiency of soot removal from the surface of cement mortar samples [98,99,100,101,102,103]. For example, Smits et al. [100] investigated the soot removal capacity of cement mortar samples coated with coatings containing four different types of TiO_2_ titanium dioxide: P25, P90, Hombikat, and E-UV, i.e., stone treatment, a product of Eoxolit based on the company’s PK-20 Precheza surrounded by nanosilica. To measure the effectiveness, they used two so far non-standardized methods of optical detection, including a colorimetric method that uses the sample’s light absorbance to calculate the degradation of soot, and a method that uses digital image processing to determine the area of soot coverage by measuring its gray. The advantages of these methods are low cost and the ability to analyze many samples simultaneously. Both methods were supplemented with gas chromatography (GC) measurements in a closed chamber in order to detect the resulting degradation products; the evidence of complete mineralization of the carbon black was the presence of one oxidation product—CO_2_. It was confirmed that the sample of mortar containing the titanium photocatalyst P25 had the best soot removal efficiency.

(c) Photoactive tests used for determining the antimicrobial activity

In order to protect construction materials against the multiplication of microorganisms, photoactive cements have begun to be tested for antimicrobial properties that may increase their life, strength, and aesthetics [61]. The disinfecting properties of the self-cleaning cement prevent the growth of pathogenic microorganisms on its surface, thus increasing its life and aesthetic durability. Moreover, by inhibiting the multiplication of microorganisms, they protect people against harmful effects on their health [4,90,91].

Exposing the self-cleaning cement to sunlight generates electron-hole pairs, which in turn generate reactive oxygen species (ROS), causing photo-oxidation of cell membrane phospholipids and other cell components in order to inhibit the growth of microorganisms [92,93,94,95,96]. So far, studies on the assessment of the antimicrobial activity of cementitious materials have not been standardized [88]. Admittedly, there is an ISO 27447 standard [97], but it does not take into account the porous structure of photoactive cement materials [88]. This may be the main reason for a much smaller number of literature reports than in the case of the previously described methods for assessing photocatalytic activity. Nevertheless, according to the current state of knowledge, the antimicrobial activity of photoactive cements is tested against bacteria [4] and fungi (molds) [61]. For example, Saini et al. investigated the antimicrobial activity of clinker cement panels with a 10% addition of calcined dolomite and a 3% addition of surface-fluorinated m-TiO_2_ against Gram-positive *Bacillus subtilis*. It has been shown that the addition of modified m-TiO_2_ to the cement inhibits the growth of the bacteria used for the test [4].

Janus et al. [96] showed that the type of photocatalyst introduced into concrete slabs is a key factor influencing the rate of bacterial removal. The antibacterial properties of n-TiO_2_ were tested by inactivating *Escherichia coli K12*. Addition of a photocatalyst in the amount of 10 wt.% led to complete inactivation of bacteria used for tests under the influence of irradiation with visible light. Commercial n-TiO_2_ P25 and doped n-TiO_2_ (12 types) were used at different temperatures, i.e., 100, 300, and 600 °C. Moreover, the doped/modified n-TiO_2_ shows better antimicrobial properties compared to the commercially available n-TiO_2_.

In turn, Hegyi et al. [62] investigated the ability to inhibit the growth of *Aspergillus niger* and *Penicillium notatum*, which are often found on building surfaces, both inside and outside buildings, and cause population diseases due to the release of aflatoxins harmful to health. They investigated the ability of mold inhibition by cement composites based on white Portland cement with the addition of n-TiO_2_. They found that the concentration of nanoparticles in the amount of 5 wt.% TiO_2_ provides a better biocidal effect than cement with the addition of 6 wt.%, which was most likely due to the difficulty of uniformly dispersing a large amount of nanoparticles in the cement matrix. Moreover, it has been shown that the resistance to mold growth differs depending on its species, by comparing the behavior of composites with the same nano-TiO_2_ content when exposed in each of the two contaminating media.

## 4. Results of Mechanical Properties of Modified Cement Mortars

There are many literature reports on the excellent properties of cement materials, such as air purification [3,7,16,17,19,23,24,104,105], self-cleaning [6,8,12,25,32,37,38,39,40,106], and self-sterilization [4,62,103]. However, despite many advantages, the wide application of photocatalytic technology in civil engineering practice is still limited [28]. For the wide use of photoactive cements, which are the basic component of photocatalytic concretes used as functional materials, they must be assessed in terms of their mechanical properties, performance, and durability during use [34].

The process influencing the mechanical properties of cements is hydration and its products, as well as the microstructural structure of the obtained composite (modification of the distribution and size of pores) [1]. It was found that the key parameter improving the performance of cement-based materials is the addition of an appropriate dose of nanomaterials [107]. Research by Nazari and Riahi [107] was conducted on the heat of hydration, cement mixtures obtained by partially replacing the binder with nano-Al_2_O_3_ at the level of 0 wt.%, 1 wt.%, 2 wt.%, 3 wt.% and 4 wt.%. They showed that the addition of nano-Al_2_O_3_ to cement slurries accelerates the hydration process at an early age. The highest rate of heat release released during the hydration process was observed for the mixture containing 3% nano-Al_2_O_3_.

Moreover, the addition of a photocatalyst to cement material may affect its workability, setting time, resistance to compression, bending, tearing, abrasion, hardness, hydrophilicity, etc. [107,108,109,110,111]. In the work of Barbhuiya et al. [112], they focused more on the microstructural properties of cementitious slurries with the addition of nano-Al_2_O_3_. They found that the microstructure of the cement-based material becomes very dense with the addition of 2% and 4% nano-Al_2_O_3_, due to the formation of larger portlandite crystals. The shift of the band in the water-related FTIR spectrum in the cement-based material with the increase of the nano-Al_2_O_3_ dose has a lower frequency. High surface activity of modified cements 1 wt.%, 2 wt.% nano-TiO_2_/S,C [113] was found, because the hydration products were deposited on the surface of nanocomposite particles, forming conglomerates with them, sealing the microstructure of the cement matrix. The results of research by Meng et al. [114] showed that after replacing cement with nano-TiO_2_, the strength of cement mortar at a young age increased significantly, probably due to an increase in hydration products. However, in later centuries, a decrease in workability and strength was noted, so it was concluded that the main cause of the increased strength at an early age may have been a change and decrease in the testicular function index.

### 4.1. Effect of Additives on Fresh Properties of Cement-Based Composite Materials—Results

(a) Effect on the workability of modified cement

An important issue concerning the use of photocatalysts in construction materials is the influence of the added additive on the mechanical and operational properties of cementitious substrates. Studies in this area have shown that the introduction of a photocatalyst affects the properties of fresh cement mortar, and the introduction of nanoparticles has a physicochemical effect [115,116].

The most often noticeable thing when preparing a mortar is to increase the amount of water needed to obtain a standard consistency [34]. Thus, the first drawback of using cement composites, which may limit the wide application by engineers, is the reduction of workability by using TiO_2_ in the mixture. A certain trend has been noticed so far. It has been reported that the greater the amount of titanium nanoparticles, the worse the workability of cement composites [115]. This was observed both in simple mixtures of cement slags and in composites with the addition of slag [115]. For example, Jimenez-Relinque et al. [6] found that the addition of 2 wt.% TiO_2_ causes a decrease in workability in the case of mortars prepared from various types of binders: OPC, CAC, fly ash, or slag. The tendency of the workability to decrease with the increase of TiO_2_ dose (from 0.5 to 5%) in simple cement mortars was also observed by other researchers [116,117,118,119,120,121,122]. Moreover, in the case of composites mixed with slag, a decrease in workability was also noticed with increasing TiO_2_ dose (e.g., 5%, 10%) [123]. This may be related to the larger specific surface area of the TiO_2_ particles, which needs more water to wet the cement particles. The studies of Sorathiya et al. [124] indicate that replacing the cement with a maximum of 1.0 wt.% TiO_2_ does not significantly affect the fluidity of the cement mortar [114,124]. Sometimes it is necessary to add a superplasticizer [123,125] to help modify the uniform strength of cement mortar with nano-TiO_2_ (NT), e.g., the addition of 0.5 wt.% of a naphthalene-based superplasticizer with a 25% water reduction coefficient [123].

(b) Effect on the initial and final setting time of modified cement

Measurements of the initial and final setting time of ordinary cement slurries and cement slurries with various additives of nanoparticles are carried out in accordance with PN-EN 196-3; 2016—Cement testing methods—Part 3: Determination of setting times and volume stability [126]. Studies by Essawa and Abd Elhaleem [106] showed that the addition of 5%, 7%, and 10% NT to a sulfate-resistant cement mixed with microsilica (MS) shortened the initial and final setting times compared to composites containing only appendix MS. Changes were also noticed in the workability of mortars; with an increase in the NT dose, the amount of water needed to obtain the desired consistency decreased, which is directly related to the smaller specific surface area of NT particles.

The tendencies of acceleration of the initial and final binding time with the increase of NT content were also observed by other researchers [116,127,128,129,130,131]. Referring to the theory, TiO_2_ nanoparticles, although chemically inert to cement components, can provide additional space for hydrated precipitation products due to their high surface area to volume ratio. Thus, they can also change the kinetics of the hydration reaction and thus the setting time [129,131].

Wang et al. [130] showed that the addition of NT reduces the initial setting time by about 40 min, from 190 min for cement without NT addition to about 150 min for cement with 5 wt% TiO_2_. On the other hand, Janus et al. [104] reported that the addition of an intermediate from the production of titanium white with the sulfate method, in the amount of 1 and 3 wt.% modified at 300 °C slightly increases the initial and final setting time compared to unmodified cement, on average by 30 min initially and the final one by about 50 min. On the other hand, for TiO_2_ modified at 600 °C, the initial one was on average 40 min longer, and the final one was about 70 min longer. It has also been found that the initial and final setting times are related to the size of the anatase crystals, and the presence of larger crystals delays the setting time significantly. This was probably due to the adsorption of water on the surface of anatase crystals [132].

(c) Effect on hydration process of modified cement

As mentioned earlier, the acceleration of the initial and final setting time of cement with nanoadditives compared to the use of a pure binder is closely related to the catalysis of the cement hydration reaction. When water is added to cement, complex hydration reactions take place with the release of heat. Scientists adhere to the principle that adding nanomaterials to cement is a way to improve its performance by modifying the hydration process [116,131], accelerating it and creating a microstructure. The changes in the hydration process are due to the small size of the incorporated nanoparticles, their large specific surface area, and high reactivity [35]. The reason for promoting the hydration of cement by nano-TiO_2_ has been partially resolved by scientists. For example, the research on the hydration of cement slurries and mortars with the addition of TiO_2_, carried out by Chen, Kou, and Poon [128], showed that the introduced additive significantly accelerated the formation rate of cement binder hydration products due to the increase in the rate of heat release and the storage function of products (filler effect additives) [33]. Moreover, the introduced TiO_2_ was inert and stable during the process [129].

As mentioned earlier, the visible effect in improving the hydration of cement mortar is the amount of heat released [33,122]. Ming-Hong Zhang emphasizes the dependence of the increase in the heat of hydration of cement for pastes in which 1–6 wt.% of TiO_2_ nanoparticles was added. With the increase in the dose of TiO_2_, the rate of cement hydration increased regardless of whether the slag was added to the mortar or not. However, in the case of mortar with the addition of 50 wt.% slag, the rate of heat released during the reaction was about 4 times lower than that of the pure mixture [122].

After loading TiO_2_ into various types of binders, an acceleration of the hydration rate and microstructure changes were observed [35], which was the main reason for the change of physical and mechanical properties of cement-based materials. Yuenyongsuwan et al. [132] proposed that the TiO_2_ crystal phase may be a key factor contributing to cement hydration. They found that rutile-rich TiO_2_ (containing 20% anatase and 80% rutile, or 50% anatase and 50% rutile) did not accelerate cement hydration, while anatase-rich TiO_2_ (containing 80% anatase and 20% rutile) did. Anatase TiO_2_ could promote cement hydration, according to the important role of potential heterogeneous nucleation sites for hydration products and the grain boundary region with dense nuclei.

Pimenta Teixeira et al. [133] showed that high temperature (40 °C and 60 °C) facilitated the hydration of cement slurry containing TiO_2_ nanoparticles. In the work of Wang et al. [130], TiO_2_ nanoparticles were added to cement mortars in the amount of 1 to 5% by weight and were subjected to reduced temperatures of 0, 5, 10, and 20 °C. XRD tests carried out on ordinary cement paste and paste with 2 wt. TiO_2_ nanoparticles at various curing temperatures at the age of 28 days showed that despite temperature changes, TiO_2_ nanoparticles did not participate in cement hydration, but only provided additional space for the attachment of hydration products such as calcium hydroxide (CH), C-S-H gels, and ettringite. Moreover, the intensity of the respective CH, C-S-H, and ettringite diffraction peaked in the cement slurry containing 2 wt. TiO_2_ nanoparticles increased compared to the peaks in the XRD spectrum of conventional cementitious paste, showing the role of TiO_2_ in accelerating the hydration process. It is also worth mentioning that as the curing temperature decreased, the amount of CH and CSH gels decreased, and the corresponding diffraction peaks decreased in intensity.

Moreover, the cement hydration process is influenced not only by the amount of the added additive, but also by its surface to volume ratio. For example, Liu et al. [134] characterized the effect of various TiO_2_ powders and nanotubes on the hydration process of cement grout. For this purpose, cement pastes, each containing 1 wt.% of the mass of cement micro-TiO_2_ (m-TiO_2_), nano-TiO_2_ (n-TiO_2_) particles, or TiO_2_ (TNT) nanotubes obtained by the hydrothermal method with a BET surface of 196 m^2^/g were used. The results obtained by X-ray diffraction (XRD) indicated that the TNT-reinforced cement paste contained the maximum amount of hydration products (Ca(OH)_2_) after 1 and 28 days of curing. This was probably due to the distinct, hollow tubular structure of TNT compared to n-TiO_2_ (45–55 m^2^/g) and m-TiO_2_ (10.89 m^2^/g), which provides more locations and nucleation sites for the formation of hydration products, which, in addition, enables the production of more products.

Wang et al. [31] found that the addition of TiO_2_ to cement 0.01%, 0.05%, and 0.10% in the form of TiO_2_ anatase hydrosol, respectively, delayed cement hydration at a young age because positively charged TiO_2_ particles carrying the COOH functional group tended to absorb on the surface of cement grains, and the growth of hydration products hindered the exchange of water and ions; what is more, this poisoned the nucleation sites.

**Figure 3:**
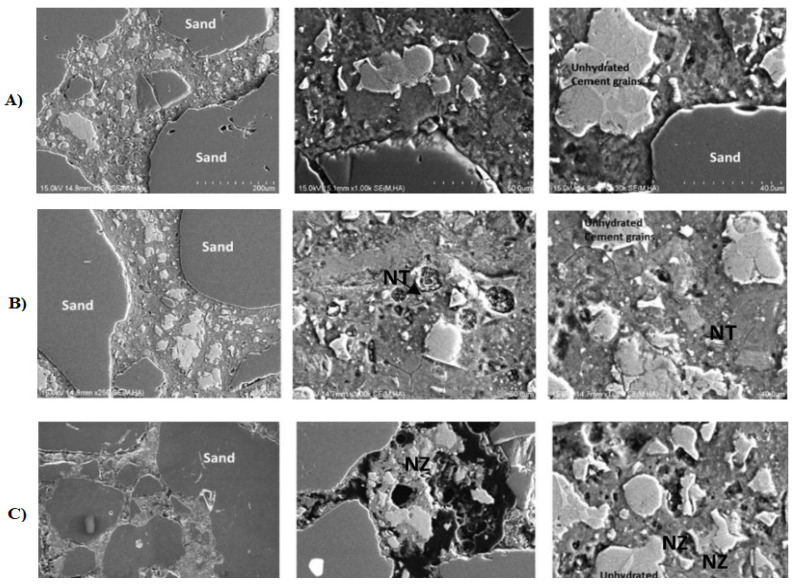
SEM photos of samples after 7 days of curing—(**A**) control sample, (**B**) cement sample with 2% nano-TiO_2_, (**C**) cement sample with 2% nano-ZnO [105] (RightsLink for Elsevier).

Different admixtures may have different effects on cement hydration behavior and microstructure formation due to their different characteristics [106,134,135]. Recent studies by Goyal et al. [105] comparing the effects of nano-TiO_2_ (NT) and nano-ZnO (NZ) on the hydration behavior of Portland cement have shown that NT accelerates hydration while NZ delays it. Based on the results of tests obtained with the XRD technique of two compared mortars, irrigated by 7 (the XRD spectrum shows the formation of CH and residual C_3_S and C_2_S) and 28 days (the XRD spectrum shows the formation of CH), it was shown that in the case of the addition of 2% NT, a significant amount of calcium hydroxide Ca(OH)_2_ (CH) is formed, whereas in the presence of 2% NZ, a small amount of CH is formed. The delaying effect of NZ is due to the formation of amorphous zinc hydroxide [136,137]. Moreover, it was previously proven that the delaying effect of the amphoteric effect of ZnO is due to the fact that it affects the products of hydration by reacting with CH to form CaZn_2_(OH)_6_ · 2H_2_O, which covers the surface of the cement particles [137]. Thermogravimetric analysis confirmed the information that NT provides more nucleation sites for hydration, which in turn speeds up the process [135]. Moreover, the SEM analysis of the mortar without nano-additive (Figure 3A) shows the formation of CH at the cement grain boundary with the formation of fine CSH and a few coarse-grained non-hydrated cement grains. In the case of cement with 2% NZ (Figure 3B), CH formation occurs at the cement grain boundary with very fine CSH formation, and compared to cement with 2% NZ (Figure 3C), a greater amount of hydration products was observed [105]. Moreover, they compared the hydration heat of cement mortars without nano-additive and 1%, 1.5%, and 2% NT or NZ addition at 25 °C and 35 °C. The results showed that on the calorimetric curve in the case of NT addition, the peak associated with hydration of silicate phases appeared much faster and increased with increasing temperature when compared to the case of pure cement. On the other hand, in the case of NZ, the hydration delay rate was significant and had little effect on the temperature. The delay rate also increased with an increase in NZ doses, while in the case of HT, rehydration occurred with an increase in the dose and temperature [105].

Zhu et al. [109] reported that the addition of 0.04% MXene with a multi-layer structure improved the early hydration of the cement-based material as well as the early strength. Its operation was to ensure the regular distribution of ettringite in the node and the formation of a network connection structure in the process of crystal growth. However, it has been found that the multilayer structure is not able to fully improve the mechanical properties.

The same research group reported that hydrated calcium silicate (C-S-H) gel formation is enhanced when delaminated MXene (Ti_3_C_2_) is mixed with cement grout. The introduced additive increases the amount of hydration products, thanks to which the structure of the hydrated cements was denser, especially when the additive was 0.01% by weight. Moreover, calorimetric studies showed that delaminated MXene inhibited the early hydration process, and after about 10–13 h, the exothermic heat of hydration was faster compared to the control sample. It was concluded that the reason for the variability in the hydration rate was due to the layered structure of MXene, which caused the water to be absorbed through the interstices between the MXene layers, which led to a reduction in water quantity and the possibility of participating in the hydration process. Finally, it is worth emphasizing that this type of nanomaterial favored cement hydration, but had no effect on the hydration products, i.e., its role was to fill and nucleate.

The addition of a different type of photocatalyst to the cement hydration process was also investigated by Liu et al. [135]. Based on XRD and hydration heat results, it was found that adding Bi_2_WO_6_ bismuth tungstate to the cement mix hardly influences the hydration process due to its inert properties. The optimal dose of 3D hierarchical Bi_2_WO_6_ was 15 wt%. Still, when dosage was increased to beyond 15%, the hydration process was deterred and hydration heat decreased.

On the other hand, loading the cement paste (0%, 0.1%, 0.5%, 1%, and 2 wt.%) with polymeric carbon nitride (PCN) suspension and maintaining hydration at various doses was studied by Yang et al. [29]. The results of the research reported that the inclusion of PCN particles may promote the early hydration of tricalcium silicate and result in a shortening of the setting time of cement mixtures, as evidenced by the increase in the heat of hydration. However, after about 48 h of irrigation, the heat of hydration decreased along with the increase in the amount of PCN. The reference paste showed the highest 68 h heat of hydration of about 259.67 J/g, then this value dropped to about 253.46 J/g and 250.68 J/g with 0.5% and 2% PCN addition, respectively, which indicates that the hydration of the clinker at a later date can be lowered by adding PCN. Moreover, it has been reported that PCN particles can play the role of both micro-fillers, improving the microstructure and reducing porosity, and can hinder the diffusion of ions and water in cement systems.

### 4.2. Effect of Additives on the Properties of Hardened-Cement-Based Composite Materials—Results

Photoactive cement composites must also meet the mechanical requirements for building materials. In order to determine the mechanical properties of cements, many tests are performed, such as, for example, bending and compressive strength tests according to PN-EN 196-1: 2016—Cement testing methods—Part I: Determination of strength [138].

The previous chapter mentioned the work of Wang et al. [130], where titanium nanoparticles were added to cement mortars in the amount of 1 to 5 wt.% and were subjected to reduced temperatures of 0, 5, 10, and 20 °C. The bending and compressive strength tests carried out after 3, 7, 28, and 56 days of hardening showed that despite the temperature changes, a rapid increase in both the compressive strength and the bending strength was observed with the increase in the amount of nano-titanium oxide to 2 wt.%. On the other hand, with higher titanium oxide contents (3–5 wt.%), the growth rate slowed down. The influence of TiO_2_ nanoparticles on the mortar strength was very clear. As is known, it is closely related to the amount, uniformity, and density of the hydrated product, mainly hydrated calcium silicate (C-S-H) gels, from the hydration of tricalcium silicate (C_3_S) and dicalcium silicate (C_2_S). A positive effect of pore filler in C-S-H gels was observed; moreover, thanks to a large specific surface, TiO_2_ nanoparticles can act as a core for the precipitation of hydration products [139]. In addition, the formation of bonds between each other and C-S-H gels, correspondingly improves strength. It was found that the lowered temperature negatively affects the mechanical parameters as it slows down the hydration process, mainly due to a delay in the production of C-S-H gels. It is also visible from the point of view of microstructural changes. In the case of the cement-based mortar, the pore volume differed between the mortar without TiO_2_ and the mortar with TiO_2_. Four types of pores were distinguished in the starting material: harmless pores (<20 nm), less harmful pores (20–50 nm), harmful pores (50–200 nm), and multiharm pores (>200 nm). Up to 2 weights of the % of titanium added to the cement matrix increased the compressive strength. At the same time, for a sample with 2 wt.%, the overall porosity decreased by 12.1%, 11.5%, 10.1%, and 7.3% at 20 °C, 10 °C, 5 °C, and 0 °C, respectively. There was also an increase in the volume of harmless and non-harmful pores, as well as a decrease in harmful pores and multiharm pores. The cement-based mortar showed a decrease in mechanical strength as the additive content increased, but the reduction was relatively small. This was explained by the appearance of a set of low-malignant pores combined with the disappearance of high-damage pores. The reason why the mechanical strength decreased so significantly in the case of low temperatures was the increased number of harmful and high-damage pores and the reduction of the volume of harmless and less harmful pores, which proves the negative influence of low temperature on the development of the pore structure.

Simultaneously with the increase in the amount of TiO_2_ nanoparticles used in the mixture, the porosity decreases. Bautista-Gutierrez and his team [140] and Lucas et al. [14] confirm the possibility of reducing the total porosity through changes taking place at the microstructural level as a result of the addition of TiO_2_ nanoparticles [14,34,139]. Moreover, the use of larger amounts of nanoparticles reduces the compressive and bending strength of concrete [140]. Singh et al. [141] confirm this position as a consequence of the heterogeneous dispersion of nanoparticles in the cement matrix, recommending the incorporation method with ultrasonic dispersion [111]. Moreover, the excess of nano-TiO_2_ is often the cause of some internal defects that reduce the durability of the material [34,142]. In Tyukavkina et al. [3], in order to evenly distribute the particles in the volume of the cement matrix, the titanium dioxide powders were subjected to ultrasonic dispersion in a saturated solution of limestone, and also in the presence of surfactants, such as sodium hexametaphosphate in the amount of 0.1% of the cement mass. It was found that the optimal content of the additive dispersed in the limestone solution was 1 wt.%, as it was characterized by the maximum strength. The preheat treatment conditions of the powder do not significantly affect the strength of the cement. The strength of the cement stone was found to increase by 22% (drying at 75 °C) and 25% with the addition of TiO_2_ (drying at 250 °C).

Other scientists also investigated the effect of the amount of nanoadditives introduced on the mechanical properties of cement mortars and concretes. Feng et al. [143] found that at room temperature the microstructure of the cement paste became denser, the number of microcracks and defects decreased with the inclusion of 0.1–1.5 wt.% of TiO_2_ nanoparticles, and the best dispersion and improvement in flexural strength were observed for the dose of 1 wt.%. Moreover, they identified three types of nanomodification mechanisms induced by nano-TiO_2_ in the cement matrix. First, it acted as activating nuclei, producing needle-shaped hydration products that fill the micropores. Second, the formation of a homogeneous, dense calcium silicate hydrate (CSH) gel was facilitated. Third, the particles aided in the permanent distribution of the hydration products throughout the cement matrix [143]. Chunping et al. [144] report an improvement in the mechanical strength of high-performance concrete and a reduction in capillary porosity of concrete by adding 1% TiO_2_ nanoparticles. Mohseni et al. found that the introduction of TiO_2_, Al_2_O_3_, or SiO_2_ nanoparticles in the amount of 1, 3, and 5 wt.% of the binder increased the compressive strength of the self-compacting mortar containing a complementary cement additive—25% addition of fly ash in relation to the total mass of the mixture at ambient temperature, and with the increase in TiO_2_ content, the compressive strength also showed an increasing trend and reached its peak on day 90 [145]. On the other hand, the addition of up to 4% of TiO_2_ particles increases the compressive strength of self-compacting concrete, while above 4% causes the opposite effect due to the limited growth space of hydration products [142]. A similar trend was observed by Zhao et al. [146], where the compressive strength after adding 10 wt.% TiO_2_ to the cement matrix decreased by 12%. In addition, some authors have observed a higher increase in compressive strength at a younger age (1, 3, and 7 days) than at an older age (28 days). For example, according to Meng et al. [123], the addition of 5% and 10% nano-TiO_2_ P25 improved the strength after 1 day by 46% and 47%, respectively, but after 28 days, a reduction of 6% and 9% was observed, respectively, compared to the control sample. In the case of modification of the cement mix in the amount of 10 wt.% of the slag powder, the compressive strength after 28 days increased by 15% and 7%, respectively. It is also worth mentioning the dependence noticed in studies where different amounts of water were used in relation to the binder and different doses of TiO_2_. With a water to binder ratio (w/b) of 0.5 at a dose of 5 wt.%, an increase in compressive strength was observed at a young age, and a decrease was observed after 28 days. In turn, the 10% additive increased compressive strength after 1 and 7 days, but decreased it after 3 and 28 days. Overall, at the ratio w/b = 0.4, an increase in compressive strength was observed with the increase in the content of TiO_2_ nanoparticles, and in the case of w/b = 0.5 and 0.6, an opposite trend was observed [33,115].

Ma et al. [147] investigated the effect of nano-TiO_2_ (NT) on the strength of hardened mortars. The bending and tensile strength of cement-based materials with different doses of TiO_2_ was tested. The results showed that the tensile and flexural strength increased with increasing NT content to 3 wt.%. The addition of 3% NT improved the compressive strength by 19.2% [148]. In turn, the influence of TiO_2_ particle size has also been identified by scientists. This cement paste, which contained 5 nm particles, showed a better compressive strength compared to the size of 25 nm. The optimal value was obtained for an NT size of 5 nm at 0.5 wt.%, with a water/cement ratio of 0.3. This mixture showed an 18.7% increase in compressive strength after 7 days and a 24.35% increase after 28 days [149].

When testing new photocatalytic cement materials, the mechanical properties were often assessed on the basis of bending strength values. In order to present the trends in the effects, a table with the results of several studies is briefly attached (Table 3). In order to obtain comparative results, in each case, the effect of the analysis of the reference material and the material are shown with an exemplary dose of photocatalysts of various types, but the main focus is on TiO_2_. First of all, TiO_2_ photocatalyst added a relatively small amount, on average up to 4% by weight. It increased the bending strength of cementitious materials. The addition of blast furnace slag, epoxy resin, and fly ash to the cement mixture had a positive effect. The mechanical properties measured in flexural strength varied with the photocatalyst used, but typically improved from about 10% to as much as about 40% after 28 days of curing. Overall, however, the increase did not exceed 13–27% after 28 days or 13–31% after 90 days. The exception is 365 days, where Hernandez et al. [42] recorded an average decrease in flexural strength ranging from about 9 to 13%. Based on the literature, optimal levels of nano-TiO_2_ replacement are 1–4 wt.%. The improvement of the properties of nanocomposites was also achieved by combining the advantages of the individual properties of TiO_2_ (self-cleaning and bactericidal) and SiO_2_ (important mechanical properties). The use of 3% TiO_2_-SiO_2_ improves the bending strength of powdered concrete by 74.9%, while 5% improves the bending strength by 87% [150]. In total, 3 wt.% addition TiO_2_-SiO_2_ of the nanocomposite has improved the mechanophysical properties thanks to the increased CSH and compact microstructure, and on the other hand, it has bactericidal properties [69]. The nanocomposite modifies the cement hydration properties more effectively due to the better dispersion of nano-TiO_2_ in the nanoparticles TiO_2_-SiO_2_ and the pozzolanic effect of SiO_2_ on the surface of the nanoparticles core/coating, and also advantageously reduces the pore volume [151]. The use of TiO_2_-SiO_2_ nanocomposite with a specific surface area greater than 300 m^2^/g allows the reduction of the amount of additive by 0.1 wt.% up to 0.5 wt.%. Moreover, it was found that the optimal temperature of the nanocomposite calcination was 800 °C, thanks to the demonstration of maximum photocatalytic activity, reduced porosity, water absorption, and abrasive losses. The increase in the compressive strength of the cement paste after 1 day of hardening is 26–29%, after 3 days 42–49%, after 28 days 38–41%, after 180 days 20–26%. [152].

**Figure 4:**
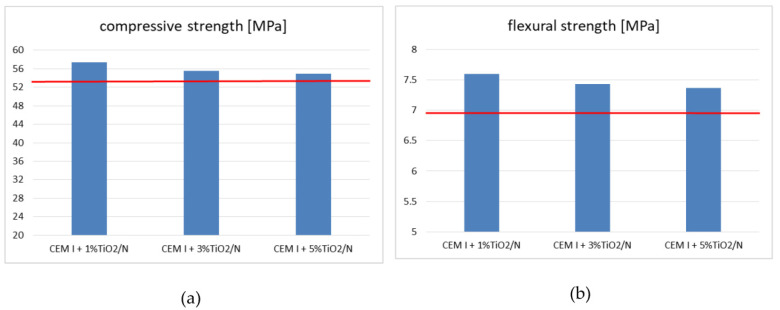
(**a**) Compressive and (**b**) flexural strength of cement mortar with the addition of 1, 3,, and 5 wt.% of photocatalyst TiO_2_/N. In the red line, the compressive strength (53 MPa) and flexural strength (6.92 MPa) of control sample are presented [23].

A positive effect on the mechanical properties is also caused by the doping of TiO_2_ with non-metals such as nitrogen, carbon, or sulfur. Compressive strength of the mortar with a content of 2.0 wt.% TiO_2_/S,C after 28 days increased by 75% compared to the control sample. In turn, the bending strength of such a mortar increased by 44% after 28 days [113]. In the studies by Janus et al. [23], the doping of titanium dioxide with nitrogen improved both the compressive and bending strength in the case of 1–5 wt.%, and this increase decreased with the increase of the dose of modified TiO_2_ (Figure 4).

In recent years, other types of photocatalysts have become increasingly popular in many modifications. Zhu et al. [109], after adding 0.01 wt. of delaminated MXene (Ti_3_C_2_), noted an increase in compressive strength by 20.2%, 45.2%, 32.6%, and 27.8%, respectively, compared to the control sample after 1, 3, 7, and 28 days. On the other hand, when the dosing of the MXene content increased from 0.01 wt.%. to 0.07 wt.%, the compressive strength was adversely reduced. Moreira et al. [25] noted a slight decrease in compressive strength after 28 days of curing, only as a result of replacing the 2% weight of cement with Nb_2_O_5_ or red mug. Another example of an alternative to replacing nanotitanium oxide is the addition of polymeric carbon nitride. The compressive strength of the mortar increased to 0.5 wt.%. With a further increase in the dose of PCN to 1% and 2%, the compressive strength of PCN-modified pastes decreased to 30.8 MPa and 26.0 MPa after 3 days and 65.7 MPa and 60.1 MPa after 28 days, which can be attributed to increasing volume of harmful pores and porosity of the cement matrix. With the increase in the maturing age from 1 day to 28 days, a drastic increase in the photocatalytic efficiency of PCN-modified pastes was observed at higher doses of PCN (≥1% of cement). The paste with the addition of 2% showed the highest photocatalytic efficiency of NOx removal after 28 days, by 122.5% higher than the paste added 2% in 1 day [29].

### 4.3. Influence of Introduced Additives on Carbonation of Cement Materials 

The inevitable, very slow, natural process of carbonation may lead to changes in the microstructure of the cement paste and in the composition of the pore solution, which may affect the performance properties of TiO_2_-modified cement materials. Proper information on structures constructed of such materials is obtained after several weeks or months, when carbonation changes the surface (Table 4) [155,156].

Rao et al. [155] applied self-compacting mortars containing 30 wt.% addition of fly ash. Water absorption after 28 days was found to increase with the addition of both nano-SiO_2_ (NS) and nano-TiO_2_ (NT) compared to the reference mortars (1:1–6.2% and 1:2–4.1%). NS blends showed higher water absorption values by immersion regardless of the degree of incorporation. This was directly related to the difficulty in dispersing nanosilica, as a result of which there is a likelihood of a less dense and homogeneous microstructure with greater porosity available for water, which was also evident in the reduction in mechanical strength. High-density mortars contain 0.5 wt.%, 0.75 wt.%, and 1 wt.%. NT is associated with the lower porosity available to water, which is also the main path for CO_2_ penetration. These samples showed no carbonation effect after 91 days of exposure in an accelerated carbonation chamber, and their compressive strength increased with age and decreased with the addition ratio of nanomaterials [155]. In general, mortars with a higher sand content (1:2) showed reduced resistance to carbonation and water absorption, which was consistent with the previous research by Huang et al. [157], which showed that CO_2_ gas penetrates the concrete surface through the sand-cement gap [157]. This means that increasing the volume of sand in concrete also increases CO_2_ penetration [158]. Duan et al. [156] noted an improvement in carbonation resistance by 77%, 62%, and 42% for fluidized bed fly ash geopolymer paste samples containing 1%, 3%, and 5% NT, respectively, after 180 days of exposure.

**Table 4:** Examples of research on the effect of carbonation of photocatalytic cement mortars.

Author	Material	Conditions	Photocatalyst Dose (wt.%)	Time of Exposure to Conditions in the Chamber	Results
Rao et al. [155]	Mortars with the addition of 30 wt.% fly ash, binder: sand ratio 1:1 and 1:2	5 ± 1% CO_2_, RH = 60 ± 5% T = 23 ± 3 °C	0.5; 0.75, 1% nano-TiO_2_ (NT) or 0.75%, 1.5%, 3% nano-SiO_2_ (NS)	At 14, 28, 56, and 91 days, the samples were taken from the chamber, broken into four parts, and the depth of carbonation was measured using the colorimetric method (with 0.1% phenolphthalein content)	1: 1 blends with 0.5 wt.%, 0.75 wt.%, and 1 wt.%. NT and 0.75% NS show total resistance to carbonation. Mixtures with nano-TiO_2_ generally showed a lower carbonation depth than blends with nano-SiO_2_. Similarly, mortars from the 1: 1 family showed a lower depth of carbonation than the mortars from the 1: 2 family.
Duan et al. [156]	Geopolymer paste based on fly ash activated in an alkaline sodium silicate solution	20% CO_2_, RH = 65 ± 5% T = 24 ± 5 °C	1, 3, 5% n-TiO_2_	At 3, 7, 28, 90, and 180 days, the depth of carbonation was measured along the exposed surface of the split specimens 40 mm long at 12 points using the phenolphthalein spray test	The improvement of the resistance to carbonation was observed only after 28 days, after 180 days, the sample with 1% TiO_2_ showed the highest resistance
Hernandez et al. [42]	Cement mortar	Normal carbonation	Addition of P25 in the amount of 5 and 10 wt.% in the surface layer	Determined with a 1% solution of phenolphthalein in ethanol after 28 days and 365 days	No significant carbonation was observed after 28 days, despite the detection of Ca(OH)_2_ by thermal analysis. Carbonation was more significant after 365 days, although mortars with/ without an additive of TiO_2_ were affected to the same extent.
Diamanti et al. [159]	Cement mortar w/c = 0.52 or w/c = 0.69	After 3 days of curing, the samples were moved to the carbonation room: 4% CO_2_, RH = 65% T = 20 °C	P25 addition in the amount of 2.5 and 5 wt.%	Determined with a solution of phenolphthalein with a concentration of 1% in ethanol after 28 days and 70 days in four points	An increase in the depth of carbonation with an increase in the w/c proportion, the addition of titanium dioxide caused a slight increase in the depth of carbonation, for example, in mixtures with a w/c of 0.69, after 70 days of exposure, the average depth of carbonation increased from about 9 mm to 11 mm and 11, 5 mm in concrete with content of 2.5 and 5% compared to cement TiO_2_.

**Figure 5:**
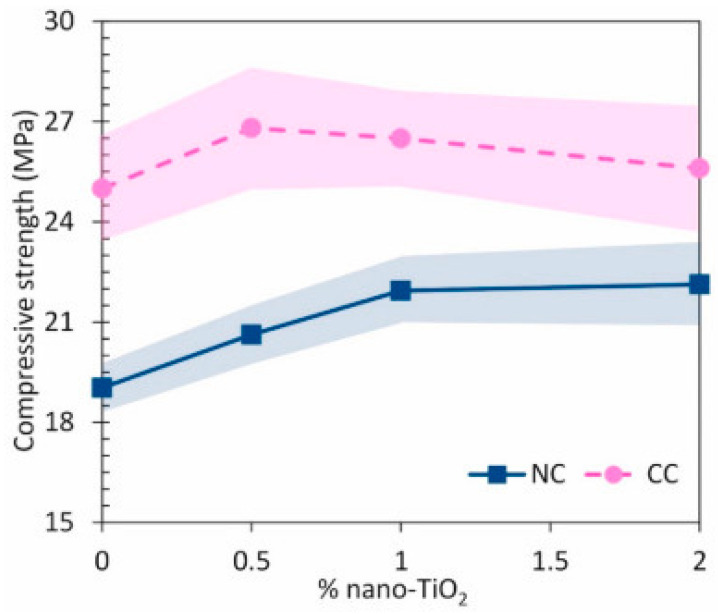
Change in compressive strength of normally and CO_2_-hardened cement pastes depending on the used dose of nano-TiO_2_ [160] (RightsLink for Elsevier).

Recent scientific studies have shown a positive effect on composite materials subjected to accelerated hardening [50]. Moreover, the influence of the carbonation process on cement composites is often considered both in terms of the induced structural changes and mechanical strength. It is known that TiO_2_ nanoparticles promote the uptake of CO_2_ during accelerated carbonation, which helps to reduce porosity, and this in turn improves the microstructure of cement composites. Moreover, in addition to increasing the absorption of CO_2_ and improving the microstructure, this process also improves the properties of cement materials, e.g., compressive strength and durability. Moro et al. [160] tested samples of cement mortars with different percentages of nano-TiO_2_ (0, 0.5, 1, 2 wt.%) subjected to CO_2_ (CC) and normal (NC) curing. XRD tests showed that CC-cured as opposed to NC-cured samples showed a significant degree of carbonation of calcium hydroxide (CH). The SEM analysis showed the microstructure densification due to the formation of calcium carbonate, with a higher molar volume than calcium hydroxide, while the samples with the addition of nano-TiO_2_ showed a more significant microstructure densification than those without. Compressive strength tests showed that for CC samples, the optimal percentage of nano-TiO_2_ (in terms of compressive strength) is lower than for normally cured samples (Figure 5). Moreover, the samples subjected to CC showed higher compressive strength regardless of the applied dose. The higher the percentage of nano-TiO_2_, the lower the initial porosity prior to the CC process, and therefore the lower the maximum potential improvement in compressive strength. This is important, as carbonation is also responsible for reducing porosity. In addition, a high reduction in porosity at a very young age may result in retention of hydration due to the lack of space for the growth of hydration products [160].

Researchers believe that changes caused by carbonation affect not only the durability of the material, but also its photocatalytic activity. Moreover, the amount of literature examining the effect of CO_2_ sequestration on changes in photocatalytic efficiency is small. On the one hand, researchers observed reduced photocatalytic activity due to carbonation on laboratory mortars, which was attributed to clogging of surface pores and, consequently, screening of exposed TiO_2_ by carbonate deposits [161,162]. Diamanti and other researchers found that the addition of 2.5 and 5 wt.% TiO2 accelerated the carbonation process; moreover, a slight increase in the depth of carbonation was noted with an increase in the water: binder ratio (w/c = 0.52 and w/c = 0.69). For the samples cured with CO_2_ for 28 and 70 days, a decrease in the final Rhodamine B precoloration was observed in all cases; however, it was still above 50%. A lower decrease in photoactivity after 70 days was noted for samples with higher content of TiO_2_ [73]. In recent studies, Moro et al. [50] compared the self-cleaning ability of cement mortars cured conventionally and in a CO_2_ environment at an early age with the addition of NT in the amount of 0.5, 1, and 2 wt.%. It was found that the self-cleaning activity increased with the dose increase, and the use of slag cement in the amount of 30 wt.% and curing with CO_2_ had a synergistic effect on cement slurries with nano-TiO_2_ in terms of reducing the porosity of the surface of the cement paste, positively influencing self-cleaning. The authors recommend a combination of the use of slag cement and CO_2_ hardening to produce photocatalytic cement composites with a lower percentage of nanoparticles up to 2 wt.%.

### 4.4. Influence of TiO_2_ on Abrasion Resistance

One of the most common forms of degradation of concrete structures is surface abrasion, which can compromise the safety of the structure and increase repair and maintenance costs. In order to minimize maintenance problems and to increase the usability of the structure, it is important that the structure is abrasion-resistant. This is closely related to the compressive strength of the abrasion surface. The influence of the presence of TiO_2_ in concrete on the abrasion resistance was investigated by Li et al. [121]. It was observed that the abrasion resistance of concrete containing nanoparticles increased with increasing compressive strength, and the relationship turned out to be linear [33]. Measurements were made after 28 days of curing in a standard humid room at 20 °C. The test results showed that the abrasion resistance of concretes containing nanoparticles improved significantly. Abrasion resistance of concrete containing 1 wt.% of nano-TiO_2_ increased by 180% for the area ratio. However, the effectiveness of TiO_2_ in increasing the abrasion resistance increased with the decrease in the photocatalyst content (5 wt.% < 3 wt.% < 1 wt.%). The abrasion resistance tests are especially important in the case of methods for depositing TiO_2_ particles on the surface of products. In Guo et al. [35], in order to assess the abrasion resistance of samples sprayed with TiO_2_ (suspension in ethanol) and mixed with 5% n-TiO_2_ P25 (Degussa), an abrasive action simulating severe abrasion conditions for pedestrians was applied. After 500-fold abrasion exposure, the SEM-EDX results showed that for the samples obtained by intercalation, the TiO_2_ content in the surface layer decreased from 2.8 wt.% to 2.0 wt.%. As for the SP sprayed samples, a significantly greater loss of TiO_2_ particles was observed, dropping from 37.5 wt.% to 12.2 wt.%. However, the results showed that the TiO_2_ content remaining on the surface of the samples sprayed with the slurry was still significantly higher than on the surface of the samples obtained by intercalation. The SP samples retained a much higher NOx removal efficiency than the samples mixed with 5% P25. It is also worth mentioning that the SP samples also showed a higher NOx removal rate before the abrasion test under different test conditions such as the following: using different flow rates, as well as initial NO concentrations, different intensity of UV radiation, different light sources (five types) as well as relative humidity, where the peak value of NOx degradation occurred at the relative humidity reaching the peak of 25% [35].

Hassan et al. [43] assessed the abrasion and wear resistance of a cement coating (w/c = 0.6) containing 3% or 5% TiO_2_ with a thickness of 10 mm applied to a concrete (substrate) layer using two types of test: accelerated load test (LWT) and rotary abrasion (RA). The measured rut depth with the wheel load tester (LWT) was minimal (less than 1 mm), indicating that the wear resistance of the surface was not affected by the application of the coating. Based on the results of the EDS analysis, it was found that the relative concentration of Ti on the used samples did not change significantly compared to the original samples, which was reflected in the photocatalytic activity tests. Namely, consumption of the samples with 5% TiO_2_ slightly decreased the NO removal efficiency of the coating from 26.9% to 22.4% for the RA samples and 23.4% for the LWT samples. In contrast, the consumption of the samples with 3% TiO_2_ slightly improved the NO removal efficiency from 18.0% to 21.4% for the RA samples and 24.8% for the LWT samples.

## 5. Examples of Investments with the Use of Photoactive Composite Materials

Photocatalytic cement products are increasingly used in the construction sector. The development of innovative, sustainable technologies for the improvement of the environment is undoubtedly an absolute need of the whole world. The use of the phenomenon of photocatalysis in the production of building materials is particularly advantageous from an ecological point of view; what is more, it fits well with the principles of sustainable construction. Many studies have shown that the skillful use of a photocatalyst makes surfaces capable of removing various hazardous and toxic compounds from the surrounding atmosphere, as well as making them capable of self-cleaning. They are also beneficial because nowadays it is difficult to limit transport and communication, which are the main source of pollution. Moreover, paved surfaces (such as highways, roads, runways, car parks, and sidewalks) typically account for 30 to 60% of developed urban areas, so they are the widest possible surface for the distribution of photocatalysts. In addition, they can be transformed during periodic maintenance or repair work. As mentioned in the introduction, Japan was the first country to introduce photoactive cement to the market [155,163,164]. Then, it also found its application in Europe [33,165]. Mitsubishi Materials Corporation and Italcementi S.p.A [33] have a patent for the production of paving stones from cement containing titanium dioxide. It is also worth mentioning the Japanese company TOTO Inc., a pioneer in the production of self-cleaning tiles based on TiO_2_, which, according to researchers Fujishima and Zhang [166], had been used in more than 5000 buildings in Japan by 2003. Moreover, the company’s report shows that after about 5 years, buildings covered with glass with TiO_2_ admixture remain clean for up to 20 years, while buildings covered with ordinary glass require general cleaning [163].

Examples of buildings using photocatalyst technology are presented below. The wide range of applications includes vertical elements such as panels and facade cladding, as well as horizontal elements such as concrete walkways. The photocatalytic properties in concrete are ensured by the addition of a nanometric catalyst to the cement matrix. However, it is also worth mentioning here the research focusing on the transformation of the product from a laboratory scale to the real scale, which must take into account many parameters determining the final efficiency of the obtained material in removing environmental pollutants [33]. It is influenced by many environmental factors, such as latitude, temperature, humidity, type of soil, amount of rain, the way in which rainwater washes the building, wind speed, its direction, UV wavelength range, etc. [33,167,168,169,170].

**Figure 6:**
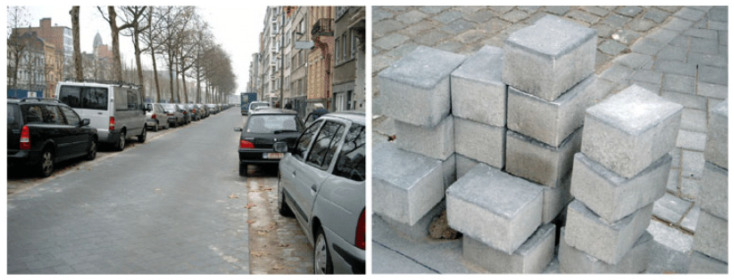
View of a parking lane with a photocatalytic paving stone in Antwerp [165].

In addition, it is a key requirement that the new products retain the long-lasting effect of self-cleaning and degradation of atmospheric pollutants, and ensure continuous contact of the photocatalyst with gaseous pollutants, especially in the case of materials exposed to abrasion and dirt. Researchers Boonen and Beeldens [165] performed laboratory and pilot scale studies. Overall, the photocatalytic concrete samples were found to be active, and the NOx reduction efficiency increased with longer contact time, lower relative humidity, and higher incident light intensity. The results obtained on the laboratory scale were converted to the real scale. In the years 2004–2005, parking lanes in the streets of the Belgian city of Antwerp were built of 10,000 m^2^ of pavement blocks (Figure 6). TiO_2_ was used only in the wearing course of concrete cubes. Multiple measurements have confirmed their effectiveness after more than 5 years of using the cubes in the field. A reduction in efficiency was observed due to the deposition of nitrate on the surface; however, the original efficiency could be regained by cleaning the surface [165]. A similar relationship was also noticed in studies conducted on a street in Bergamo, Italy [171]. Namely, the NOx reduction was significantly higher on the days when the paving stones were cleaned; however, compared to the unmodified section of the asphalt road, the NOx reduction values for the two-layer photocatalytic concrete paving stone were as high as 60%.

Also in Poland, the first pavements and bicycle paths were made of photocatalytic cobblestones, among others in Zielona Góra, Nowa Sól, Chorula, and Boguchwała [52]. The research group of Witkowski [52] and others investigated the durability of the photocatalytic properties of pavement blocks removed from the bicycle path located on the main street in Zielona Góra. The results showed that the ability to reduce NOx concentration in the air was strongly dependent on the UV light source. Contrary to the studies by Boonen and Beeldens [165], the purity level of the photoactive surface did not significantly affect the NO reduction result. However, it was found that the main factor influencing the efficiency is the source of UV radiation. After cleaning the sample and using a more intense UV light source (300 W), a 45% reduction of NO was observed [52].

**Figure 7:**
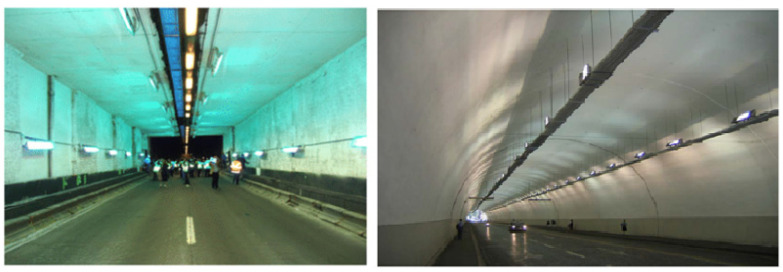
Tunnels after renovation: Leopold II tunnel in Brussels (**left**) [165] and “Umberto I’’ in Rome (**right**) [172] (RightsLink for Elsevier).

Implementation efforts aimed at demonstrating the usefulness of photocatalytic building materials were also undertaken in Belgium [165]. In this case, during the 2011 renovation, a coating of photocatalytic cement materials approximately 100 m long was applied to the side walls and roof of the Leopold II Tunnel in Brussels (Figure 7). Special lighting emitting UV light was used to activate the photocatalysis reaction. The results of measurements carried out during the field campaign did not show a significant reduction in pollution due to the deactivation of the photocatalytic surface by dust and soot, too low irradiation, and too high wind speed [169,170]. A similar solution was used to activate the photocatalysis mechanism earlier in the “Umberto I” road tunnel in Rome, 350 m long (2007), where photocatalytic cement paints applied by spraying were used for renovation—gray to a height of 1.80 m and white above (Figure 7). The results of the measurement campaign of nitrogen oxides concentration measurements showed a reduction of NO by 25% and NO_2_ by 19% [172].

Air pollution caused by excessive NOx emissions is a serious problem not only in tunnels, but also near roads and heavy traffic areas, and is a particular problem for people who spend a lot of time in these places. Thus, field tests were conducted not only for tunnels, but also for horizontal applications. Witkowski et al. [167] presented the results of field studies on the use of photocatalytic cobblestones in Warsaw to reduce NOx pollution. The studies on a larger scale were preceded by laboratory experiments in which the effectiveness of NOx reduction was proven in accordance with the UNI 11247 standard [173]. The test area was located at the Daszyńskiego roundabout in Warsaw (Poland) and next to the office building. Concentrations of NO, NO_2_, and NOx were measured on a pavement with conventional concrete paving stones and on a photocatalytic paving stone (catalyst embedded only in a five-millimeter surface layer) both 400 mm above the pavement surface. The mean daily reduction was 31%, which was above the threshold for the target’s large-scale use (15%) [167]. An important step was the researchers proposing an innovative research method that used light conditions and gas flow that most faithfully reflected real conditions. Moreover, the test configuration [51,167] reduced the negative influence of wind and insufficient concentration of pollutants, as in Folli et al. [174]. In Denmark, on the central street of Copenhagen, near the Central Station, one hundred meters of pavement is made of unmodified concrete blocks and the other is made of 100 m of modified titanium dioxide. Annual measurements showed a low value (below 40 ppb) of the average daily NO concentration in the paved area with concrete elements containing TiO_2_. In addition, some seasonal variability was recorded. Namely, as the relative humidity increased, the conversion of NO decreased as a result of competition between water and NO for catalytic sites. On the other hand, with increasing temperature, NO conversion increased due to the greater diffusivity of gaseous pollutants towards the photocatalytic surface. NO reduction peaked greater than 45% at the summer solstice.

Testing of the photocatalytic material in real conditions was also carried out by Ballari and Brouwers [175]. The NOx concentration was measured on a street in Hengelo, the Netherlands, with a concrete pavement containing TiO_2_ and a control street depending on the weather conditions. Overall, the NOx concentration was 19% lower compared to the unmodified system. However, when only in the afternoons or under conditions of high irradiation and low relative humidity, this value was 28% or 45% lower, respectively, than the control route. Under ideal weather conditions, the NOx removal efficiency was maximum 45%. The proposed solutions are promising techniques for reducing the number of air pollutants, especially in locations with high levels of pollution, such as busy canyon streets or road tunnels.

Field studies by Huang et al. [176] suggested that in rush hours, even higher deNOx efficiency was achieved over pavements coated with carbon nitride/TiO_2_ composite graphite hydrosol (gC_3_N_4_/TiO_2_). Moreover, the NO removal rate was found to be 70.7% in the morning and afternoon, i.e., under conditions of weak solar radiation.

In addition to the ability to clean the air, an important issue is also to ensure the durability of the aesthetics of the building through the decomposition of organic and inorganic pollutants deposited on the surfaces of building materials. This is extremely important for white cement buildings. In terms of aesthetic durability, experimental studies have shown that the maintenance of white or colored (in the case of a colored matrix) color is not affected by the amount of nanoparticle addition. TiO_2_ particles are characterized by a high degree of whiteness, but their relatively even weight distribution does not affect the possibility of dyeing with conventional pigments. After dyeing the composite mass, no time loss was observed due to the photocatalytic activity of TiO_2_ nanoparticles [115].

The self-cleaning of concrete surfaces results from the photocatalytic and superhydrophilic properties of nanocrystalline titanium dioxide. A prime example of the use of self-cleaning concrete is the church designed by Richard Meier, “Dives in Misericordia” in Rome, commissioned in 2003 [168]. It was found that during the six years of monitoring, only a slight difference was observed between the outer and inner white walls [33]. However, in 2018, Cardellicchio [168] reports an accelerated decomposition of external walls manifested by black spots all over the concrete. Despite the lack of self-cleaning properties, the case study made it possible to test the durability and effectiveness of this specific application of TiO_2_ under real-world conditions. Despite the poor appearance of the structure and the present surface condition, the results of chemical analysis confirmed the presence of titanium dioxide in the form of anatase particles [168]. Studies have shown that the necessary hydrophilic effect is limited by two main factors. The first is related to the composition of pozzolana dust in Rome, which cannot be oxidized, and its combination with the products of erosion creates a patina that inactivates the surface even more. The second is the abrasive action of rainwater on the spherical surface, which increases the surface roughness of the concrete and thus increases the bonding of rain dust particles with the concrete. Researchers highlight the fact that, due to the chemical composition and abrasive action of pozzolana dust, the self-cleaning properties of nanostructured titanium particles may be at risk in countries where the soil is predominantly volcanic or where desert ash precipitation is frequent. These environmental factors may increase the frequency of washing for the maintenance of concrete panels, detracting from the aesthetic, economic, and environmental benefits of using a self-cleaning material [168].

## 6. Conclusions and Future Prospects

1. The presented research shows that the prepared concretes show photocatalytic activity and can clean the air, e.g., removing nitrogen oxides or volatile organic compounds. What is more, they have the ability to degrade soot and microorganisms, maintaining the lasting appearance of the building during its use.
2. Photoactive cements used in the production of concrete products are mainly activated by UV radiation; scientists tried to find a photocatalyst active in visible light. Additional studies are needed to demonstrate durability in the field of photocatalysts other than TiO_2_.
3. The procedure for introducing the photocatalytic active ingredient influences the surface concentration of the catalytic active sites, giving preference to the surface coating method over the bulk application of mortar when discussing only the photocatalytic efficiency, but without changing the nature of the active sites. However, the introduction of the active phase of the catalyst to the mass, even with a lower but still acceptable photocatalytic activity, results in a more stable system in terms of mechanical properties of the surface and CSH distribution. Given the need to balance the different photocatalyst performance requirements with the expected overall product yields, the incorporation of the active catalyst phase into the bulk appears to be more promising for sustainable long-term applications.

However, based on the literature, it was found that the surface coating method in the production of photocatalytic materials has a greater range, thanks to the possibility of renovating existing buildings. Although the prices of commercial specialty cements are much higher than the unmodified ones, they offset the costs so that renovation is much less frequent than with the use of ordinary materials.

4. The issue that cannot be ignored in the research is the place, terrain conditions, and climate where the photocatalytic building, road, pavement, etc. will be located.
5. The presented research has shown that when designing a photocatalytic mortar, the type of binder, final texture, and microstructure of the material should be carefully selected to meet the performance requirements. Research is also needed to check the performance of the technology in the field.

## Figures and Tables

**Table 1:** Methods of obtaining photoactive cements.

Type of Method	Short Description
(A) Incorporation method.	Adding a photocatalyst during the manufacture of cement; cement replacement by mass with photocatalytic TiO_2_ particles (micro- or nanosized).
(B) Photocatalyst coating technology—cement coating with a thin layer of TiO_2_ materials with (I) or without (II) a separation layer.	Creating coatings (generally 200 nm thick) by sprinkling with powder, applying paints, enamels, TiO_2_ suspensions, or special composites, e.g., TiO_2_/ZnAl. The coatings are applied by techniques such as direct painting on the surface of the cement matrix—by wet coating method; by immersion, spraying, spray pyrolysis, electrodeposition, or chemical vapor deposition (CVD).
(C) Addition of the photocatalyst only to the top of the cement mortar layer.	Addition of TiO_2_ or modified TiO_2_ to the surface layer; lower layer is unmodified concrete; the top part of concrete consists of cement with photocatalyst.

**Table 2:** Parameters of the photocatalytic efficiency test according to ISO 22197-1.

Gas concentration [ppm]	1.0
Gas flow rate [dm^3^/min]	3.0
Duration of the test	5 h
Radiation intensity [mV/cm^2^]	1
Sample surface [cm^2^]	50
Pretreatment of the sample	16–24 h of UV irradiation with a power of ≥1.5 mW/cm^2^ without gas flow
Temperature	25 °C
Analytical method	NOx chemiluminescence analyzer

**Table 3:** Flexural strength of different types of building materials.

Author and Used Photocatalytic Material	Building Material	Age (Days)	Exemplary Dose (wt.%)	Flexural Strength (MPa)	
				Photocatalytic Sample—Description of the Change or Value of the Bending Strength Respective Dose	Reference Sample
Wang et al. [130] n-TiO_2_ (10–25 nm, 200 m^2^/g)	Cement mortar	56	1, 2, 3	12.3, 13.8, 13.6	10.7
Meng et al. [114] nano-TiO_2_ (20–50 nm) 39.91 m^2^/g	Cement paste, cement + fly ash 20 wt.%	30 min during ball milling	1	increasing bending strength by 37.74%	9.99
Han et al. [151] nano-SiO_2_ coated by TiO_2_	Reactive concrete powder, w/c = 0.3	28	1, 3, 5	9.60, 12.45, 14.38	6.69
Hernandez [42] Aeroxide TiO_2_ P25	White and grey cement mortar w/c in layer =1.3	28	5, 10	G-8.21 (5%) 8.29 (10%) W-8.70 i 7.53	G = 9.05 W = 8.65
		365		G-11.20 (5%) 10.91 (10%) W-10.69 i 10.32	G = 12.26 W = 12.01
Yang et al. [153] TiO_2_ (20–100 nm)	slag paste activated with alkali	3	0.5	7.71	6.17
		7		12.46	10
		28		17.32	12.58
Zhang et al. [122] n-TiO_2_ or n-SiO_2_	Concrete	28	1	n-TiO_2_: 6.02 n-SiO_2_: 5.69	5.46
Nazari i Riahi [118] nano-TiO_2_ (15 nm) 155 m^2^/g	Cement mortar, 1 wt.% superplasticizer, w/c = 0.4	28	1–5	increase in bending strength to 4 wt.%, the highest value after 28 days	4.2
Nazari i Riahi [129] nano-TiO_2_ (15 nm)	Concrete with 15, 30.45.60 wt.% replacement with blast furnace slag	28	1–4	up to 3 wt.% TiO_2_ and 45 wt.% slag bending strength increased	4.2
		90			5.6
Feng et al. [143] nano-TiO_2_ (20–50 nm)	Cement paste, w/c = 0.4	28	0.1	12.05	11.53
			0.5	12.45	
			1	12.48	
			5	12.30	
Lucas et al. [14] P25 (85% anatase, 15% rutile) (21 nm)	Cement, cement-lime, gypsum, or gypsum-lime mortar	28	0.5–5	Cement and lime-cement mortars show a loss of bending strength in addition to more than 1 wt.%; the gypsum plaster showed a 60% reduction in strength at 0.5% wt., which indicates a greater difficulty for incorporation of nanoparticles	8.0
Guo et al. [10] nano-TiO_2_ (100 nm)	Cement mortar modified with epoxy resin TiO_2_	7, 28	0, 1, 3 or 5 wt.%. in admixture with pure epoxy resin	increase with increasing dose and curing period	8.8 9.5
Ma et al. [147] smoky nano-TiO_2_ 50 m^2^/g	Cement mortar and concrete	3 28 90	1–5	increase with increasing dose up to 4 wt.% and curing period	2.82 4.42 6.28
Rahim and Nair [154] nano-TiO_2_, nano-Al_2_O_3_ or nano-SiO_2_	Cement mortar partially replaced by fly ash and blast furnace slag	28	2, 3, 4, 5, 6	After 28 days of hardening to 4 wt.%. nano-TiO_2_, 3 wt.% Al_2_O_3_, and nano-SiO_2_, an increase in flexural strength was observed	9.0
Sikora et al. [69] TiO_2_ P25 (21 nm) n-SiO_2_ mSiO_2_/TiO_2_ (mesoporous silica nanospheres modified with titanium dioxide)	Cement mortar	28	3	TiO_2_ P25: 7.0 n-SiO_2_: 7.0 mSiO_2_/TiO_2_: 7.3	7.1
Ng et al. [116] TiO_2_ (15 nm) NT Fe_2_O_3_ (20–40 nm) NF SiO_2_ (20–30 nm) NS	Cement mortar with an admixture of 30 wt.% fly ash w/b = 0.485	28	1,3,5	The increase is 19%, 11%, and 10%, respectively, in the NS, NT, and NF samples compared to the control sample. The optimal dose is 3 wt.% for each additive in terms of mechanical properties	NS- circa 4.8 NT- 4.9 NF- circa 4.4
Cerro-Prada et al. [80] TiO_2_/100% Anataz (20–30 nm)	Cement mortar	1, 7, 28, 90	0.1, 0.2, 0.5, 1—without and with replacement of cement	For samples with cement replacement, in the early and middle age of the mortar (2, 7, and 28), slightly reduced strength is obtained for the substitute content of nano-TiO_2_ of 0.1%, 0.5%, and 1%. By replacing TiO_2_ in cement with 0.2%, however, a slight improvement in bending strength (13.7%) is achieved in the long term. In the case of the mortar prepared with the addition of TiO_2_ without cement replacement, no improvement can be clearly observed for the TiO_2_ content of 0.2%, 0.5%, and 1%	[80]

## References

[1] Hamidi F., Aslani F. (2019). TiO_2_-based Photocatalytic Cementitious Composites: Materials, Properties, Influential Parameters, and Assessment Techniques. Nanomaterials.

[2] Carmona-Quiroga P., Martínez-Ramírez S., Viles H. (2018). Efficiency and durability of a self-cleaning coating on concrete and stones under both natural and artificial ageing trials. Appl. Surf. Sci..

[3] Tyukavkina V.V., Gerasimova L.G., Semushin V.V. (2019). Properties of Compositions Based on Cement and Modified Nanodispersed Titanium Dioxide. Inorg. Mater. Appl. Res..

[4] Saini A., Ratan J.K. (2022). Formulation and evaluation of surface-fluorinated microsized-TiO_2_ based self-cleaning cement: Characterization, self-cleaning, depollution and antimicrobial study. Chem. Pap..

[5] Chen J., Poon C.-S. (2009). Photocatalytic construction and building materials: From fundamentals to applications. Build. Environ..

[6] Jimenez-Relinque E., Rodriguez-Garcia J., Castillo A., Castellote M. (2015). Characteristics and efficiency of photocatalytic cementitious materials: Type of binder, roughness and microstructure. Cem. Concr. Res..

[7] Jin Q., Hordern S.L., Tang Y., Kurtis K.E. (2021). NO_x_ sequestration by calcium aluminate cementitious materials. Cem. Concr. Res..

[8] Baltes L., Patachia S., Tierean M., Ekincioglu O., Ozkul H.M. (2018). Photoactive glazed polymer-cement composite. Appl. Surf. Sci..

[9] Zhao P., Wang H., Wang S., Du P., Lu L., Cheng X. (2018). Assessment of Nano-TiO_2_ Enhanced Performance for Photocatalytic Polymer-Sulphoaluminate Cement Composite Coating. J. Inorg. Organomet. Polym. Mater..

[10] Guo S.-Y., Zhang X., Ren J., Chen J.-Z., Zhao T.-J., Li T.-W., Zhang L. (2020). Preparation of TiO_2_/epoxy resin composite and its effect on mechanical and bonding properties of OPC mortars. Constr. Build. Mater..

[11] Binas V., Papadaki D., Maggos T., Katsanaki A., Kiriakidis G. (2018). Study of innovative photocatalytic cement based coatings: The effect of supporting materials. Constr. Build. Mater..

[12] Folli A., Jakobsen U.H., Guerrini G.L., Macphee D. (2009). Rhodamine B Discolouration on TiO_2_ in the Cement Environment: A Look at Fundamental Aspects of the Self-cleaning Effect in Concretes. J. Adv. Oxid. Technol..

[13] Ratan J.K., Saini A., Verma P. (2018). Microsized-titanium dioxide based self-cleaning cement: Incorporation of calcined dolomite for enhancement of photocatalytic activity. Mater. Res. Express.

[14] Lucas S., Ferreira V., de Aguiar J.B. (2012). Incorporation of titanium dioxide nanoparticles in mortars—Influence of microstructure in the hardened state properties and photocatalytic activity. Cem. Concr. Res..

[15] Folli A. (2010). TiO_2_ Photocatalysis in Portland Cement Systems: Fundamentals of Self-Cleaning Effect and Air Pollution Mitigation. Ph.D. Thesis.

[16] Chen J., Poon C.-S. (2009). Photocatalytic Cementitious Materials: Influence of the Microstructure of Cement Paste on Photocatalytic Pollution Degradation. Environ. Sci. Technol..

[17] Guo M.-Z., Poon C.S. (2018). Superior photocatalytic NOx removal of cementitious materials prepared with white cement over ordinary Portland cement and the underlying mechanisms. Cem. Concr. Compos..

[18] Cassar L., Pepe C., Tognon G., Guerrini G.L., Amadelli R. white cement for architectural concrete, possessing photocatalytic properties. Proceedings of the 11th International Congress on the Chemistry of Cement.

[19] Lee B.Y., Jayapalan A.R., Bergin M.H., Kurtis K.E. (2014). Photocatalytic cement exposed to nitrogen oxides: Effect of oxidation and binding. Cem. Concr. Res..

[20] Bianchi C., Gatto S., Nucci S., Cerrato G., Capucci V. (2013). Self-cleaning measurements on tiles manufactured with micro-sized photoactive TiO_2_. Adv. Mater. Res..

[21] Quagliarini E., Bondioli F., Goffredo G.B., Cordoni C., Munafò P. (2012). Self-cleaning and de-polluting stone surfaces: TiO_2_ nanoparticles for limestone. Constr. Build. Mater..

[22] Amor F., Diouri A., Ellouzi I., Ouanji F. (2018). Development of Zn-Al-Ti mixed oxides-modified cement phases for surface photocatalytic performance. Case Stud. Constr. Mater..

[23] Janus M., Mądraszewski S., Zając K., Kusiak-Nejman E., Morawski A.W., Stephan D. (2019). Photocatalytic Activity and Mechanical Properties of Cements Modified with TiO_2_/N. Materials.

[24] Janus M., Zając K., Zatorska J., Kusiak-Nejman E., Czyżewski A., Morawski A.W. (2015). Cementitious Plates Containing TiO_2_-N,C Photocatalysts for NO_x_ Degradation. J. Adv. Oxid. Technol..

[25] Moreira M.A., Heitmann A.P., Bezerra A.C., Patrício P.S., de Oliveira L.C., Castro C.S., de Souza P.P. (2020). Photocatalytic performance of cementitious materials with addition of red mud and Nb_2_O_5_ particles. Constr. Build. Mater..

[26] Wang S., Ang H., Tadé M. (2008). Novel applications of red mud as coagulant, adsorbent and catalyst for environmentally benign processes. Chemosphere.

[27] Klauber C., Gräfe M., Power G. (2011). Bauxite residue issues: II. options for residue utilization. Hydrometallurgy.

[28] Geng Z., Zhang L., Wang J., Yu Y., Zhang G., Cheng X., Wang D. (2021). BiOBr Precursor Solutions Modified Cement Paste: The Photocatalytic Performance and Effects. Crystals.

[29] Yang Y., Yan Z., Zheng L., Yang S., Su W., Li B., Ji T. (2022). Interaction between composition and microstructure of cement paste and polymeric carbon nitride. Constr. Build. Mater..

[30] Wang D., Hou P., Zhang L., Xie N., Yang P., Cheng X. (2018). Photocatalytic activities and chemically-bonded mechanism of SiO_2_@ TiO_2_ nanocomposites coated cement-based materials. Mater. Res. Bull..

[31] Wang Z., Yu Q., Gauvin F., Feng P., Qianping R., Brouwers H. (2020). Nanodispersed TiO_2_ hydrosol modified Portland cement paste: The underlying role of hydration on self-cleaning mechanisms. Cem. Concr. Res..

[32] Lee J.W., Lee S.H., Jang Y.I., Park H.M. (2021). Evaluation of Reducing NO and SO_2_ Concentration in Nano SiO_2_-TiO_2_ Photocatalytic Concrete Blocks. Materials.

[33] Janus M., Zając K. (2016). Concretes with Photocatalytic Activity, High Performance Concrete Technology and Applications.

[34] Mendoza C., Valle A., Castellote M., Bahamonde A., Faraldos M. (2014). TiO_2_ and TiO_2_–SiO_2_ coated cement: Comparison of mechanic and photocatalytic properties. Appl. Catal. B Environ..

[35] Guo M.-Z., Ling T.-C., Poon C.S. (2017). Photocatalytic NO x degradation of concrete surface layers intermixed and spray-coated with nano-TiO 2: Influence of experimental factors. Cem. Concr. Compos..

[36] Vulic T., Rudic O., Vucetic S., Lazar D., Ranogajec J. (2015). Photocatalytic activity and stability of TiO_2_/ZnAl layered double hydroxide based coatings on mortar substrates. Cem. Concr. Compos..

[37] Wang D., Hou P., Zhang L., Yang P., Cheng X. (2017). Photocatalytic and hydrophobic activity of cement-based materials from benzyl-terminated-TiO_2_ spheres with core-shell structures. Constr. Build. Mater..

[38] Carrascosa L.A.M., Zarzuela R., Badreldin N., Mosquera M.J. (2020). A Simple, Long-Lasting Treatment for Concrete by Combining Hydrophobic Performance with a Photoinduced Superhydrophilic Surface for Easy Removal of Oil Pollutants. ACS Appl. Mater. Interfaces.

[39] Feng S., Li F. (2021). Photocatalytic dyes degradation on suspended and cement paste immobilized TiO_2_/g-C_3_N_4_ under simulated solar light. J. Environ. Chem. Eng..

[40] Carp O., Huisman C.L., Reller A. (2004). Photoinduced reactivity of titanium dioxide. Prog. Solid State Chem..

[41] Zhong W., Wang D., Jiang C., Lu X., Zhang L., Cheng X. (2020). Investigation of visible light catalysis of a graphite-nitride-silica composite material and its cement surface treatment. Crystals.

[42] Hernández-Rodríguez M.J., Rodríguez R.S., Darias R., Díaz O.G., Luzardo J.M.P., Rodríguez J.M.D., Melián E.P. (2019). Effect of TiO_2_ Addition on Mortars: Characterization and Photoactivity. Appl. Sci..

[43] Hassan M., Dylla H., Mohammad L.N., Rupnow T. (2010). Evaluation of the durability of titanium dioxide photocatalyst coating for concrete pavement. Constr. Build. Mater..

[44] Huang M., Yang Z., Lu L., Xu J., Wang W., Yang C. (2022). The Preparation of g-C_3_N_4_/CoAl-LDH Nanocomposites and Their Depollution Performances in Cement Mortars under UV-Visible Light. Catalysts.

[45] Jimenez-Relinque E., Castellote M. (2019). Quick assessment of the photocatalytic activity of TiO_2_ construction materials by nitroblue tetrazolium (NBT) ink. Constr. Build. Mater..

[46] Baglioni P., Cassar L., Hashimoto K., Filippone F., Mattioli G., Bonapasta A.A., Bianchi C.L., Ardizzone S., Cappelletti G., Pirola C. (2007). International RILEM Symposium on Photocatalysis, Environment and Construction Materials-TDP 2007.

[47] Evans P., Mantke S., Mills A., Robinson A., Sheel D. (2007). A comparative study of three techniques for determining photocatalytic activity. J. Photochem. Photobiol. A Chem..

[48] Bengtsson N., Castellote M. (2010). Photocatalytic Activity for NO Degradation by Construction Materials: Parametric Study andMultivariable Correlations. J. Adv. Oxid. Technol..

[49] Jimenez-Relinque E., Castellote M. (2015). Quantification of hydroxyl radicals on cementitious materials by fluorescence spectrophotometry as a method to assess the photocatalytic activity. Cem. Concr. Res..

[50] Moro C., Francioso V., Lopez-Arias M., Velay-Lizancos M. (2022). Modification of self-cleaning activity on cement pastes containing nano-TiO_2_ due to CO_2_ curing. Constr. Build. Mater..

[51] Shayegan Z., Lee C.-S., Haghighat F. (2018). TiO_2_ photocatalyst for removal of volatile organic compounds in gas phase—A review. Chem. Eng. J..

[52] Witkowski H., Jackiewicz-Rek W., Chilmon K., Jarosławski J., Tryfon-Bojarska A., Gąsiński A. (2019). Air Purification Performance of Photocatalytic Concrete Paving Blocks after Seven Years of Service. Appl. Sci..

[53] Mills A., Elouali S. (2015). The nitric oxide ISO photocatalytic reactor system: Measurement of NO_x_ removal activity and capacity. J. Photochem. Photobiol. A Chem..

[54] Diamanti M.V., Luongo N., Massari S., Spagnolo S.L., Daniotti B., Pedeferri M. (2021). Durability of self-cleaning cement-based materials. Constr. Build. Mater..

[55] (2016). Fine Ceramics (Advanced Ceramics, Advanced Technical Ceramics)—Test Method for Air-Purification Performance of Semiconducting Photocatalytic Materials—Part 1: Removal of Nitric Oxide.

[56] Şahin O., Bay S., Ilcan H., Yıldırım G., Şahmaran M. (2020). Influence of mixing methods on the NOx reduction capability and electrical properties of photocatalytic cementitious systems. Cem. Concr. Compos..

[57] Seo D., Yun T.S. (2017). NOx removal rate of photocatalytic cementitious materials with TiO 2 in wet condition. Build. Environ..

[58] Mothes F., Ifang S., Gallus M., Golly B., Boréave A., Kurtenbach R., Kleffmann J., George C., Herrmann H. (2018). Bed flow photoreactor experiments to assess the photocatalytic nitrogen oxides abatement under simulated atmospheric conditions. Appl. Catal. B Environ..

[59] (2019). Fine Ceramics (Advanced Ceramics, Advanced Technical Ceramics)—Test Method for Air-Purification Performance of Semiconducting Photocatalytic Materials—Part 3: Removal of Toluene.

[60] Martinez T., Bertron A., Escadeillas G., Ringot E., Simon V. (2014). BTEX abatement by photocatalytic TiO_2_-bearing coatings applied to cement mortars. Build. Environ..

[61] Graziani L., Quagliarini E., Bondioli F., D’Orazio M. (2014). Durability of self-cleaning TiO_2_ coatings on fired clay brick façades: Effects of UV exposure and wet & dry cycles. Build. Environ..

[62] Hegyi A., Lăzărescu A.-V., Szilagyi H., Grebenişan E., Goia J., Mircea A. (2021). Influence of TiO_2_ Nanoparticles on the Resistance of Cementitious Composite Materials to the Action of Bacteria. Materials.

[63] Ratan J.K., Saini A. (2019). Enhancement of photocatalytic activity of self-cleaning cement. Mater. Lett..

[64] Wang D., Hou P., Stephan D., Huang S., Zhang L., Yang P., Cheng X. (2020). SiO_2_/TiO_2_ composite powders deposited on cement-based materials: Rhodamine B removal and the bonding mechanism. Constr. Build. Mater..

[65] Feng S., Song J., Liu F., Fu X., Guo H., Zhu J., Zeng Q., Peng X., Wang X., Ouyang Y. (2020). Photocatalytic properties, mechanical strength and durability of TiO_2_/cement composites prepared by a spraying method for removal of organic pollutants. Chemosphere.

[66] Lettieri M., Colangiuli D., Masieri M., Calia A. (2018). Field performances of nanosized TiO_2_ coated limestone for a self-cleaning building surface in an urban environment. Build. Environ..

[67] García L., Pastor J., Peña J. (2018). Self cleaning and depolluting glass reinforced concrete panels: Fabrication, optimization and durability evaluation. Constr. Build. Mater..

[68] Laplaza A., Jimenez-Relinque E., Campos J., Castellote M. (2017). Photocatalytic behavior of colored mortars containing TiO_2_ and iron oxide based pigments. Constr. Build. Mater..

[69] Sikora P., Cendrowski K., Markowska-Szczupak A., Horszczaruk E., Mijowska E. (2017). The effects of silica/titania nanocomposite on the mechanical and bactericidal properties of cement mortars. Constr. Build. Mater..

[70] Taurino R., Barbieri L., Bondioli F. (2016). Surface properties of new green building material after TiO_2_–SiO_2_ coatings deposition. Ceram. Int..

[71] Calia A., Lettieri M., Masieri M. (2016). Durability assessment of nanostructured TiO_2_ coatings applied on limestones to enhance building surface with self-cleaning ability. Build. Environ..

[72] Cohen J.D., Gallego G.A.S., Tobón J.I. (2015). Evaluation of Photocatalytic Properties of Portland Cement Blended with Titanium Oxynitride (TiO_2_−xN_y_) Nanoparticles. Coatings.

[73] Diamanti M., Del Curto B., Ormellese M., Pedeferri M. (2013). Photocatalytic and self-cleaning activity of colored mortars containing TiO_2_. Constr. Build. Mater..

[74] Le Pivert M., Poupart R., Capochichi-Gnambodoe M., Martin N., Leprince-Wang Y. (2019). Direct growth of ZnO nanowires on civil engineering materials: Smart materials for supported photodegradation. Microsyst. Nanoeng..

[75] Calvo J.G., Carballosa P., Castillo A., Revuelta D., Gutiérrez J., Castellote M. (2019). Expansive concretes with photocatalytic activity for pavements: Enhanced performance and modifications of the expansive hydrates composition. Constr. Build. Mater..

[76] Koli V.B., Mavengere S., Kim J.-S. (2019). An efficient one-pot N doped TiO_2_-SiO_2_ synthesis and its application for photocatalytic concrete. Appl. Surf. Sci..

[77] Kapridaki C., Xynidis N., Vazgiouraki E., Kallithrakas-Kontos N., Maravelaki-Kalaitzaki P. (2019). Characterization of Photoactive Fe-TiO_2_ Lime Coatings for Building Protection: The Role of Iron Content. Materials.

[78] Zanfir A.-V., Voicu G., Bădănoiu A.-I., Gogan D., Oprea O., Vasile E. (2018). Synthesis and characterization of titania-silica fume composites and their influence on the strength of self-cleaning mortar. Compos. Part B Eng..

[79] Graziani L., Quagliarini E., D’Orazio M. (2016). The role of roughness and porosity on the self-cleaning and anti-biofouling efficiency of TiO 2 -Cu and TiO 2 -Ag nanocoatings applied on fired bricks. Constr. Build. Mater..

[80] Cerro-Prada E., García-Salgado S., Quijano M., Varela F. (2018). Controlled Synthesis and Microstructural Properties of Sol-Gel TiO_2_ Nanoparticles for Photocatalytic Cement Composites. Nanomaterials.

[81] Alfieri I., Lorenzi A., Ranzenigo L., Lazzarini L., Predieri G., Lottici P.P. (2017). Synthesis and characterization of photocatalytic hydrophobic hybrid TiO_2_ -SiO_2_ coatings for building applications. Build. Environ..

[82] Li Q., Liu Q., Peng B., Chai L., Liu H. (2016). Self-cleaning performance of TiO_2_ -coating cement materials prepared based on solidification/stabilization of electrolytic manganese residue. Constr. Build. Mater..

[83] Yousefi A., Allahverdi A., Hejazi P. (2013). Effective dispersion of nano-TiO_2_ powder for enhancement of photocatalytic properties in cement mixes. Constr. Build. Mater..

[84] Werle A., de Souza M., Loh K., Ando R., John V. (2016). The performance of a self-cleaning cool cementitious surface. Energy Build..

[85] Mills A., Hepburn J., Hazafy D., O’Rourke C., Wells N., Krysa J., Baudys M., Zlamal M., Bartkova H., Hill C.E. (2014). Photocatalytic activity indicator inks for probing a wide range of surfaces. J. Photochem. Photobiol. A Chem..

[86] Mills A., O’Rourke C., Lawrie K., Elouali S. (2013). Assessment of the Activity of Photocatalytic Paint Using a Simple Smart Ink Designed for High Activity Surfaces. ACS Appl. Mater. Interfaces.

[87] Mills A., Wells N., O’Rourke C. (2014). Correlation between ΔAbs, ΔRGB (red) and stearic acid destruction rates using commercial self-cleaning glass as the photocatalyst. Catal. Today.

[88] Mills A., Hill C., Robertson P.K. (2012). Overview of the current ISO tests for photocatalytic materials. J. Photochem. Photobiol. A: Chem..

[89] Mills A. (2012). An overview of the methylene blue ISO test for assessing the activities of photocatalytic films. Appl. Catal. B Environ..

[90] Bolashikov Z., Melikov A., Bolashikov Z., Melikov A. (2009). Methods for air cleaning and protection of building occupants from airborne pathogens. Build. Environ..

[91] Wiszniewska M., Walusiak-Skorupa J., Gutarowska B., Krakowiak A., Pałczyński C. (2009). Is the risk of allergic hypersensitivity to fungi increased by indoor exposure to moulds?. Int. J. Occup. Med. Environ. Health.

[92] Özyıldız F., Güden M., Uzel A., Karaboz I., Akil O., Bulut H. (2010). Antimicrobial activity of TiO_2_-coated orthodontic ceramic brackets against Streptococcus mutans and Candida albicans. Biotechnol. Bioprocess Eng..

[93] Ganguly P., Byrne C., Breen A., Pillai S.C. (2018). Antimicrobial activity of photocatalysts: Fundamentals, mechanisms, kinetics and recent advances. Appl. Catal. B Environ..

[94] Ganji N., Allahverdi A., Naeimpoor F., Mahinroosta M. (2015). Photocatalytic effect of nano-TiO_2_ loaded cement on dye decolorization and Escherichia coli inactivation under UV irradiation. Res. Chem. Intermed..

[95] Velez-Pena E., Perez-Obando J., Pais-Ospina D., Marin-Silva D.A., Pinotti A., Canneva A., Rengifo-Herrera J.A. (2020). Self-cleaning and antimicrobial photo-induced properties under indoor lighting irradiation of chitosan films containing Mel-on/TiO_2_ composites. Appl. Surf. Sci..

[96] Janus M., Kusiak-Nejman E., Rokicka-Konieczna P., Markowska-Szczupak A., Zając K., Morawski A.W. (2019). Bacterial Inactivation on Concrete Plates Loaded with Modified TiO_2_ Photocatalysts under Visible Light Irradiation. Molecules.

[97] (2019). Fine Ceramics (Advanced Ceramics, Advanced Technical Ceramics)—Test Method for Antibacterial Activity of Semiconducting Photocatalytic Materials.

[98] Luna M., Delgado J.J., Gil M.L.A., Mosquera M.J. (2018). TiO_2_-SiO_2_ Coatings with a Low Content of AuNPs for Producing Self-Cleaning Building Materials. Nanomaterials.

[99] Pozo-Antonio J., Dionísio A. (2017). Self-cleaning property of mortars with TiO_2_ addition using real diesel exhaust soot. J. Clean. Prod..

[100] Smits M., Chan C.K., Tytgat T., Craeye B., Costarramone N., Lacombe S., Lenaerts S. (2013). Photocatalytic degradation of soot deposition: Self-cleaning effect on titanium dioxide coated cementitious materials. Chem. Eng. J..

[101] Luna M., Mosquera M.J., Vidal H., Gatica J.M. (2019). Au-TiO_2_/SiO_2_ photocatalysts for building materials: Self-cleaning and de-polluting performance. Build. Environ..

[102] Truppi A., Luna M., Petronella F., Falcicchio A., Giannini C., Comparelli R., Mosquera M.J. (2018). Photocatalytic Activity of TiO_2_/AuNRs–SiO_2_ Nanocomposites Applied to Building Materials. Coatings.

[103] Smits M., Huygh D., Craeye B., Lenaerts S. (2014). Effect of process parameters on the photocatalytic soot degradation on self-cleaning cementitious materials. Catal. Today.

[104] Janus M., Mądraszewski S., Zając K., Kusiak-Nejman E. (2020). A New Preparation Method of Cement with Photocatalytic Activity. Materials.

[105] Goyal R., Verma V.K., Singh N. (2022). Effect of nano TiO_2_ & ZnO on the hydration properties of Portland cement. Mater. Today Proc..

[106] Essawy A.A., El Aleem S.A. (2014). Physico-mechanical properties, potent adsorptive and photocatalytic efficacies of sulfate resisting cement blends containing micro silica and nano-TiO_2_. Constr. Build. Mater..

[107] Nazari A., Riahi S. (2011). RETRACTED ARTICLE: Effects of Al_2_O_3_ nanoparticles on properties of self compacting concrete with ground granulated blast furnace slag (GGBFS) as binder. Sci. China Technol. Sci..

[108] Gawande M.B., Goswami A., Asefa T., Guo H., Biradar A.V., Peng D.-L., Zboril R., Varma R.S. (2015). Core–shell nanoparticles: Synthesis and applications in catalysis and electrocatalysis. Chem. Soc. Rev..

[109] Zhu J., Li G., Feng C., Wang L., Zhang W. (2019). Effect of Delaminated MXene (Ti_3_C_2_) on the Performance of Cement Paste. J. Nanomater..

[110] Rashad A.M. (2015). A synopsis about the effect of nano-titanium dioxide on some properties of cementitious materials-a short guide for civil engineer. Rev. Adv. Mater. Sci..

[111] Hegyi A., Szilagyi H., Grebenisan E., Sandu A.V., Lăzărescu A.-V., Romila C. (2020). Influence of TiO_2_ Nanoparticles Addition on the Hydrophilicity of Cementitious Composites Surfaces. Appl. Sci..

[112] Barbhuiya S., Mukherjee S., Nikraz H. (2014). Effects of nano-Al_2_O_3_ on early-age microstructural properties of cement paste. Constr. Build. Mater..

[113] Hohol M., Sanytsky M., Kropyvnytska T., Barylyak A., Bobitski Y. (2020). The effect of sulfur-and carbon-codoped TiO_2_ nano-composite on the photocatalytic and mechanical properties of cement mortars. East.-Eur. J. Enterp. Technol..

[114] Meng J., Zhong J., Xiao H., Ou J. (2020). Interfacial design of nano-TiO_2_ modified fly ash-cement based low carbon composites. Constr. Build. Mater..

[115] Grebenişan E., Szilagyi H., Hegyi A.-C., Mircea C., Baeră C. (2019). Opportunities regarding the potential use of the self-cleaning concept within urban contemporary architecture in Romania. MATEC Web Conf..

[116] Ng D.S., Paul S.C., Anggraini V., Kong S.Y., Qureshi T.S., Rodriguez C.R., Liu Q.-F., Šavija B. (2020). Influence of SiO_2_, TiO_2_ and Fe2O_3_ nanoparticles on the properties of fly ash blended cement mortars. Constr. Build. Mater..

[117] Jalal M., Ramezanianpour A.A., Pool M.K. (2012). Effects of titanium dioxide nanopowder on rheological properties of self compacting concrete. Am. J. Sci..

[118] Nazari A., Riahi S., Riahi S., Shamekhi S.F., Khademno A. (2010). Assessment of the effects of the cement paste composite in presence TiO_2_ nanoparticles. Am. J. Sci..

[119] Salemi N., Behfarnia K., Zaree S.A. (2014). Effect of nanoparticles on frost durability of concrete. Asian J. Civ. Eng. BHRC.

[120] Li H., Zhang M.-H., Ou J.-P. (2007). Flexural fatigue performance of concrete containing nano-particles for pavement. Int. J. Fatigue.

[121] Li H., Zhang M.-H., Ou J.-P. (2006). Abrasion resistance of concrete containing nano-particles for pavement. Wear.

[122] Zhang M.-H., Li H. (2011). Pore structure and chloride permeability of concrete containing nano-particles for pavement. Constr. Build. Mater..

[123] Meng T., Yu Y., Qian X., Zhan S., Qian K. (2012). Effect of nano-TiO_2_ on the mechanical properties of cement mortar. Constr. Build. Mater..

[124] Sorathiya J., Shah S., Kacha S. (2017). Effect on addition of nano “titanium dioxide”(TiO_2_) on compressive strength of cementitious concrete. Kalpa Publ. Civ. Eng..

[125] Zhang M.H., Tanadi D., Li W. Effect of photocatalyst TiO_2_ on workability, strength, and self-cleaning efficiency of mortars for applications in tropical environment. Proceedings of the 35th Conference on Our World in Concrete & Structures.

[126] (2016). Cement Testing Methods—Part 3: Determination of Setting Times and Volume Stability.

[127] Lee B.Y. (2012). Effect of Titanium Dioxide Nanoparticles on Early Age and Long Term Properties of Cementitious Materials. Ph.D. Thesis.

[128] Chen J., Kou S.-C., Poon C.-S. (2012). Hydration and properties of nano-TiO_2_ blended cement composites. Cem. Concr. Compos..

[129] Nazari A., Riahi S., Riahi S., Shamekhi S.F., Khademno A. (2010). Improvement the mechanical properties of the cementitious composite by using TiO_2_ nanoparticles. Am. J. Sci..

[130] Wang L., Zhang H., Gao Y. (2018). Effect of TiO_2_ Nanoparticles on Physical and Mechanical Properties of Cement at Low Temperatures. Adv. Mater. Sci. Eng..

[131] Zając K., Czyżewski A., Kaszyńska M., Janus M. (2020). Combined Effect of Photocatalyst, Superplasticizer, and Glass Fiber on the Photocatalytic Activity and Technical Parameters of Gypsum. Catalysts.

[132] Yuenyongsuwan J., Sinthupinyo S., O’Rear E.A., Pongprayoon T. (2018). Hydration accelerator and photocatalyst of nanotitanium dioxide synthesized via surfactant-assisted method in cement mortar. Cem. Concr. Compos..

[133] Teixeira K.P., Rocha I.P., Carneiro L.D.S., Flores J., Dauer E.A., Ghahremaninezhad A. (2016). The Effect of Curing Temperature on the Properties of Cement Pastes Modified with TiO_2_ Nanoparticles. Materials.

[134] Liu J., Jee H., Lim M., Kim J.H., Kwon S.J., Lee K.M., Nezhad E.Z., Bae S. (2020). Photocatalytic Performance Evaluation of Titanium Dioxide Nanotube-Reinforced Cement Paste. Materials.

[135] Liu P., Gao Y., Wang F., Zhang W., Yang L., Yang J., Liu Y. (2016). Photocatalytic activity of Portland cement loaded with 3D hierarchical Bi2WO6 microspheres under visible light. Constr. Build. Mater..

[136] Behfarnia K., Azarkeivan A., Keivan A. (2013). The effects of TiO_2_ nad ZnO nanoparticles on physical and mechanical properties of normal conctere. Asian. J. Civil. Eng..

[137] Gawlicki M., Czamarska D. (1992). Effect of ZnO on the hydration of Portland cement. J. Therm. Anal..

[138] (2016). Cement Testing Methods. Part 1: Determination of Strength.

[139] Lawrence P., Cyr M., Ringot E. (2003). Mineral admixtures in mortars: Effect of inert materials on short-term hydration. Cem. Concr. Res..

[140] Bautista-Gutierrez K.P., Herrera-May A.L., Santamaría-López J.M., Honorato-Moreno A., Zamora-Castro S.A. (2019). Recent Progress in Nanomaterials for Modern Concrete Infrastructure: Advantages and Challenges. Materials.

[141] Singh L., Karade S., Bhattacharyya S., Yousuf M., Ahalawat S. (2013). Beneficial role of nanosilica in cement based materials—A review. Constr. Build. Mater..

[142] Nazari A., Riahi S. (2011). RETRACTED: TiO_2_ nanoparticles effects on physical, thermal and mechanical properties of self compacting concrete with ground granulated blast furnace slag as binder. Energy Build..

[143] Feng D., Xie N., Gong C., Leng Z., Xiao H., Li H., Shi X. (2013). Portland Cement Paste Modified by TiO_2_ Nanoparticles: A Microstructure Perspective. Ind. Eng. Chem. Res..

[144] Gu C. (2018). Effect of nano-tio₂ on the durability of ultra-high performance concrete with and without a flexural load. Ceram. Silik..

[145] Mohseni E., Miyandehi B.M., Yang J., Yazdi M.A. (2015). Single and combined effects of nano-SiO_2_, nano-Al_2_O_3_ and nano-TiO_2_ on The mechanical, rheological and durability properties of self-compacting mortar containing fly ash. Constr. Build. Mater..

[146] Zhao A., Yang J., Yang E.-H. (2015). Self-cleaning engineered cementitious composites. Cem. Concr. Compos..

[147] Ma B., Li H., Mei J., Li X., Chen F. (2015). Effects of Nano-TiO_2_ on the Toughness and Durability of Cement-Based Material. Adv. Mater. Sci. Eng..

[148] Chinthakunta R., Ravella D.P., Chand M.S.R., Yadav M.J. (2021). Performance evaluation of self-compacting concrete containing fly ash, silica fume and nano titanium oxide. Mater. Today: Proc..

[149] Sun Y., Zhang P., Guo W., Bao J., Qu C. (2020). Effect of Nano-CaCO_3_ on the Mechanical Properties and Durability of Concrete Incorporating Fly Ash. Adv. Mater. Sci. Eng..

[150] Han B., Li Z., Zhang L., Zeng S., Yu X., Han B., Ou J. (2017). Reactive powder concrete reinforced with nano SiO_2_-coated TiO_2_. Constr. Build. Mater..

[151] Sun J., Xu K., Shi C., Ma J., Li W., Shen X. (2017). Influence of core/shell TiO_2_@SiO_2_ nanoparticles on cement hydration. Constr. Build. Mater..

[152] Shchelokova E., Tyukavkina V., Tsyryatyeva A., Kasikov A. (2021). Synthesis and characterization of SiO_2_-TiO_2_ nanoparticles and their effect on the strength of self-cleaning cement composites. Constr. Build. Mater..

[153] Yang L., Jia Z., Zhang Y., Dai J. (2015). Effects of nano-TiO_2_ on strength, shrinkage and microstructure of alkali activated slag pastes. Cem. Concr. Compos..

[154] Rahim A., Nair S.R. (2016). Influence of nano-materials in high strength concrete. J. Chem. Pharm. Sci..

[155] Rao S., Silva P., de Brito J. (2015). Experimental study of the mechanical properties and durability of self-compacting mortars with nano materials (SiO_2_ and TiO_2_). Constr. Build. Mater..

[156] Duan P., Yan C., Luo W., Zhou W. (2016). Effects of adding nano-TiO _2_ on compressive strength, drying shrinkage, carbonation and microstructure of fluidized bed fly ash based geopolymer paste. Constr. Build. Mater..

[157] Huang Q., Jiang Z., Zhang W., Gu X., Dou X. (2012). Numerical analysis of the effect of coarse aggregate distribution on concrete carbonation. Constr. Build. Mater..

[158] Al Fuhaid A.F., Niaz A. (2022). Carbonation and Corrosion Problems in Reinforced Concrete Structures. Buildings.

[159] Diamanti M., Lollini F., Pedeferri M., Bertolini L. (2013). Mutual interactions between carbonation and titanium dioxide photoactivity in concrete. Build. Environ..

[160] Moro C., Francioso V., Velay-Lizancos M. (2021). Impact of nano-TiO_2_ addition on the reduction of net CO_2_ emissions of cement pastes after CO_2_ curing. Cem. Concr. Compos..

[161] Lackhoff M., Prieto X., Nestle N., Dehn F., Niessner R. (2003). Photocatalytic activity of semiconductor-modified cement—Influence of semiconductor type and cement ageing. Appl. Catal. B Environ..

[162] Sanchez F., Sobolev K. (2010). Nanotechnology in concrete—A review. Constr. Build. Mater..

[163] Fujishima A., Zhang X., Tryk D.A. (2008). TiO_2_ photocatalysis and related surface phenomena. Surf. Sci. Rep..

[164] Guo S., Wu Z., Zhao W. (2009). TiO_2_-based building materials: Above and beyond traditional applications. Sci. Bull..

[165] Boonen E., Beeldens A. (2012). Photocatalytic roads: From lab tests to real scale applications. Eur. Transp. Res. Rev..

[166] Fujishima A., Zhang X. (2006). Titanium dioxide photocatalysis: Present situation and future approaches. Comptes Rendus. Chim..

[167] Witkowski H., Jarosławski J., Tryfon-Bojarska A. (2020). Application of Photocatalytic Concrete Paving Blocks in Poland—Verification of Effectiveness of Nitric Oxides Reduction and Novel Test Method. Materials.

[168] Cardellicchio L. (2020). Building defects in new iconic structures: The technical challenge and the economic impact of restoring the Jubilee Church in Rome. Arch. Eng. Des. Manag..

[169] Boonen E., Akylas V., Barmpas F., Boréave A., Bottalico L., Cazaunau M., Chen H., Daële V., De Marco T., Doussin J.F. (2015). Construction of a photocatalytic de-polluting field site in the Leopold II tunnel in Brussels. J. Environ. Manag..

[170] George C., Beeldens A., Barmpas F., Doussin J.-F., Manganelli G., Herrmann H., Kleffmann J., Mellouki A. (2016). Impact of photocatalytic remediation of pollutants on urban air quality. Front. Environ. Sci. Eng..

[171] Guerrini G.L. (2009). Some observations regarding in–service performance Photocatalytic paving block surfaces. Betonw. Fert. Teil-Tech. BFT.

[172] Guerrini G.L. (2012). Photocatalytic performances in a city tunnel in Rome: NO_x_ monitoring results. Constr. Build. Mater..

[173] (2010). Determination of the Degradation of Nitrogen Oxides in the Air by Inorganic Photocatalytic Materials: Continuous Flow Test Method.

[174] Folli A., Strøm M., Madsen T.P., Henriksen T., Lang J., Emenius J., Klevebrant T., Nilsson Å. (2015). Field study of air purifying paving elements containing TiO_2_. Atmos. Environ..

[175] Ballari M.M., Brouwers H.J.H. (2013). Full scale demonstration of air-purifying pavement. J. Hazard. Mater..

[176] Huang Y., Zhang J., Wang Z., Liu Y., Wang P., Cao J.J., Ho W.K. (2020). g-C_3_N_4_/TiO_2_ composite film in the fabrication of a pho-to-catalytic air-purifying pavements. Sol. RRL.

